# Halotolerant bacterial endophyte *Bacillus velezensi*s CBE mediates abiotic stress tolerance with minimal transcriptional modifications in *Brachypodium distachyon*

**DOI:** 10.3389/fpls.2024.1485391

**Published:** 2025-01-10

**Authors:** Islam A. Abd El-Daim, Gareth Raynes, Narcis Fernandez-Fuentes, Sarah Hawkins, Alan Cookson, Kerrie Farrar

**Affiliations:** ^1^ Institute of Biological, Environmental and Rural Sciences (IBERS) Aberystwyth University, Aberystwyth, United Kingdom; ^2^ Department of Microbiology, Soils, Water and Environment Research Institute, Agricultural Research Centre, Giza, Egypt

**Keywords:** abiotic stress, beneficial microbes, endophytes, transcription, tolerance

## Abstract

Nitrogen and water are the primary resources limiting agricultural production worldwide. We have demonstrated the ability of a novel halotolerant bacterial endophyte, *Bacillus velezensi*s CBE, to induce osmotic stress tolerance in *Brachypodium distachyon* under nitrogen-deprived conditions. Additionally, we aimed to identify the molecular factors in plants that contribute to the beneficial effects induced by *B. velezensis* CBE in *B. distachyon*. To achieve this, we conducted transcriptomic profiling using RNA-seq on 18-day-old *B. distachyon* seedlings treated with *B. velezensis* CBE in the presence or absence of available nitrogen, with and without osmotic stress. These profiles were then compared to those obtained from *B. distachyon* treated with known plant growth-promoting bacterial strains, *Azospirillum brasilense* Cd and *Azoarcus olearius* DQS4, under the same growth conditions. We identified differentially expressed genes (DEGs) in response to the combinations of bacterial strains and stress treatments. Interestingly, only 73 transcripts showed significant differential expression in *B. velezensis* CBE-treated plants under stress conditions, compared to 1,078 DEGs in plants treated with *A. brasilense* Cd and 2,015 DEGs in *A. olearius* DQS4. Our findings suggest that the novel endophyte *B. velezensis* CBE mediates osmotic stress tolerance in *B. distachyon* through the fine-tuning of molecular mechanisms with minimal transcriptional modifications.

## 1 Introduction

Stresses such as drought, salinity, and nutrient limitations are the main limiting factors for plant growth and productivity (Fahad et al., 2017). The situation has been exacerbated by the drastic and rapid changes in global climate. For instance, drought due to water scarcity is one of the most critical issues potentially compromising global food security (Raza et al., 2019). Environmental stress factors are known to provoke complex transcriptional changes in plants, and comprehensive transcriptome profiling has led to the identification of two major categories of genes (Debnath et al., 2011). The first group includes genes that encode proteins involved in cellular homeostasis and protection from stress, such as osmolytes, chaperones, antioxidative enzymes, metabolic enzymes, and lipid-transfer proteins. The second group mainly includes kinases and transcription factors that regulate the stress signal transduction and stress-responsive gene transcription (Yoshida et al., 2014; Sewelam et al., 2016; Haak et al., 2017; Tiwari et al., 2017).

Stress tolerance in plants can be enhanced through the treatment of plants or seeds with certain natural and synthetic compounds or microorganisms (Rai et al., 2021). Plant-beneficial bacteria, commonly referred to as plant growth-promoting bacteria (PGPB), are known to be associated with several plant species (Glick, 2012). They may have different mechanisms to support plant growth and health, including the ability to mediate abiotic stress tolerance (Liu et al., 2020; Yang et al., 2009). Thus, certain PGPB strains are extensively investigated for their potential to control plant stress (Vurukonda et al., 2016; Naing et al., 2021). For instance, it was reported that *A. brasilense* can mediate abiotic stress tolerance in several plant species including salt stress in barley (Omar et al., 2009) and drought stress in wheat (Kasim et al., 2013) and maize (Curá et al., 2017). Furthermore, Naveed et al. (2014) reported the ability of *Paraburkholderia phytofirmans* PsJN to mediate drought stress tolerance in maize. *P. phytofirmans* PsJN was also found to induce salt stress tolerance in *Arabidopsis thaliana* (Pinedo et al., 2015). PGPB utilize several mechanisms to induce abiotic stress tolerance in plants (Dimkpa et al., 2009; Yang et al., 2009). PGPB can enhance plant growth directly by providing plants with nutrients such as nitrogen via nitrogen fixation or by supplying phosphorus from soil-bound phosphate (Di Benedetto et al., 2017). PGPB are known for their ability to synthesize several plant growth hormones such as auxin and cytokinins and further modulate the plant stress hormone ethylene via 1-aminocyclopropane-1-carboxylate (ACC) deaminase production (Glick, 2014).

Plant responses to PGPB are not well characterized; however, they are thought to be dependent on numerous factors, including plant genotype and the associated bacterial strain (Vandana et al., 2020). Reports suggest that the plant PGBP crosstalk is complex and can manifest in significant metabolic and physiological responses (Abd El-Daim et al., 2019). The metabolic and physiological changes caused by PGPB are expected to be driven by complex molecular and signaling tuning through plant transcriptional regulation (Abd El-Daim et al., 2018; Backer et al., 2018; Abd El-Daim et al., 2019). PGPB appear to program relatively similar plant molecular factors to mediate abiotic stress tolerance in plants, e.g., classical defense response pathway genes such as the well-characterized plant transcription factor WRKY, ABA-responsive MYB, and ethylene-responsive factors ERFs are known to be modulated by PGPB treatment (Bruto et al., 2014; Abd El-Daim et al., 2018). However, due to the vast diversity of PGPB, it is also possible that certain PGPB strains will have different impacts on such broad transcriptional pathways. For instance, *Enterobacter* sp. strain C7 alleviated drought stress by reducing the expression of ethylene-responsive genes in tomatoes (Ibort et al., 2018). In contrast, *B. megaterium* inoculation mediated drought stress tolerance by inducing the expression of ethylene response genes in tomatoes (Ibort et al., 2018). Furthermore, certain PGPB strains may induce abiotic stress tolerance by molecular tuning of more specific genes. The PGPB *Dietzia natronolimnaea* strain STR1 confers salt tolerance to wheat via increased expression of the *SOS4* pyridoxal kinase, which controls N^+^ and K^+^ balance through adjusting ion transporter activity (Rosier et al., 2018). Moreover, Pinedo et al. (2015) reported that the ability of *P. phytofirmans* PsJN to mediate salt stress tolerance in *A. thaliana* is related to the accumulation of proline and transcription of *RD29A* and *RD29B* genes.

*B. velezensis* CBE is a novel endophyte isolated from the stem of the *Calluna vulgaris* plant growing on the coastal path near Aberystwyth, Wales, where it experienced regular salt spray. The strain is highly tolerant toward salt stress and further showed the potential of improving *B. distachyon* vegetative growth under salt stress conditions (**Supplementary Figure S1**). The present study aimed to elucidate the plant molecular factors involved in *B. velezensis* CBE’s ability to mediate abiotic stress tolerance in *B. distachyon.* Differentially expressed genes were identified by whole-transcriptome shotgun sequencing (RNA-Seq) analysis on total RNA extracted from *B. distachyon* seedlings treated with novel hallo-tolerant endophyte *B. velezensis* CBE and grown under osmotic stress and nitrogen-free conditions. Furthermore, we aimed to test whether the plant molecular modulations induced by *B. velezensis* CBE are common transcriptional regulations involved in PGPB-mediated abiotic stress tolerance in Brachypodium. To achieve this, we compared the transcriptome profile of *B. distachyon* treated with *B. velezensis* CBE with the profiles obtained for *B. distachyon* treated with known PGPB strains *A. brasilense* Cd and *A. olearius* DQS4.

## 2 Materials and methods

### 2.1 *Bacillus velezensis* CBE isolation and characterization

The strain, along with others, was isolated from a surface-sterilized stem of *Calluna vulgaris*, collected along the coastal path between Clarach and Aberystwyth (52°25′53.9″ 4°04′42.2″W) (**Supplementary Figure S1A**). The sterilized stem was divided into three parts: one left whole, and two ground in 1 ml of sterile water, with one of the ground samples further diluted 1^10^ in sterile water. These three sample subsets (whole, ground, and diluted ground tissue) were used to inoculate nutrient agar plates supplemented with either rock salt or sea salt (SAXA Rock Salt and Cornish Sea Salt) at final concentrations of 3.5% w/v and 1% w/v, respectively, to selectively isolate halotolerant endophytes. The plates were sealed with parafilm and incubated for 4 weeks at ambient lab temperature, allowing slower-growing, less-characterized endophytes to emerge under natural day/night cycles. Colonies of interest were picked from the agar plates and streaked onto fresh nutrient agar plates for further identification and characterization.

The bacterial isolate was identified by amplifying and sequencing the 16S rRNA gene using the primers 8F (AGAGTTTGATCCTGGCTCAG) and 1378R (CGGTGTGTACAAGGCCCGGGAACG). The amplified PCR product was sequenced via Sanger sequencing using an ABI 3730 DNA analyzer at Aberystwyth University. The resulting sequence was analyzed in CHROMAS, and a BLAST search was performed using the NIH Bacterial 16S reference library. The strain used in this study was identified as *Bacillus velezensis*. The sequence has been deposited in GenBank under accession number PQ498196.

The strain’s salt tolerance was characterized on nutrient agar plates supplemented with 1 to 2 M NaCl (methodology used is described as **Supplementary Information**). CBE demonstrated the ability to survive at the highest tested salt concentration for up to 186 h (**Supplementary Table S1**). The ability of *B. velezensis* CBE was found to improve the vegetative biomass of salt-stressed *B. distachyon* (**Supplementary Figure S1B**).

### 2.2 Plant growth, bacterial and abiotic stress treatment

*Brachypodium distachyon* Bd21 seeds were sourced from the National Plant Phenomics Centre (NPPC), Aberystwyth, UK. Seeds were dehusked in order to increase germination efficiency and uniformity. Dehusked seeds were submerged in 70% ethanol for 5 min, rinsed in sterile dH_2_O, and submerged in 4% bleach solution for 5 min before being rinsed again in sterile dH_2_O. The final wash was plated on nutrient agar plates to determine the sterility of the seed surfaces. Surface sterilized seeds were transferred to Petri dishes containing presterilized filter paper moistened with 5 ml of sterile dH_2_O. The germination plates were sealed with micropore tape and placed into darkness for one day at room temperature, followed by 3 days on the windowsill in full day/night light cycle conditions until the seed coating had split and the root had begun to emerge.

*A. brasilense* Cd and *A. olearius* DQS4 were obtained from Dr. Euan James at the James Hutton Institute, Invergowrie, Dundee, UK. *A. brasilense* Cd, a rhizosphere strain originally isolated from mangrove roots by Holguin et al. (1992), is known for its ability to fix nitrogen and mediate salt stress tolerance in plants (Bacilio et al., 2004). *A. olearius* DQS4, a nitrogen-fixing bacterium isolated by Chen et al. (2013), is genetically and phenotypically similar to the model grass endophyte *A. olearius* BH72 (Faoro et al., 2017; Raittz et al., 2021). It is recognized for its ability to endophytically colonize and promote plant growth across various species (Faoro et al., 2017).

*B. velezensis* CBE, *A. brasilense* Cd, and *A. olearius* DQS4 were cultured separately on full-strength tryptic soya broth (TSB) media (Sigma Aldrich, Germany) overnight at 28°C with 180 RPM shaking. Four days old *B. distachyon* Bd21 seedlings were bacterially treated by soaking in TSB based bacterial solution consisting of 10^7^ ml^−1^ of the desired bacterial strain for 20 min at room temperature. Seedlings reserved for both negative and positive controls were treated by sterile TSB media.

Four bacterial treated or untreated seedlings were transferred to sterile plastic containers filled with 50 ml of pure semisolid autoclaved water agar or supplemented with either 3 mM ammonium nitrate, 5% polyethylene glycol (PEG 8000), or both. The containers were transferred to a plant growth cabinet and maintained in a controlled environment at 22/16°C (day/night), with a 16/8-h photoperiod, light intensity of 450 µmol m^−2^ s^−1^, and 80% humidity. The sealed containers supported plant growth for up to 14 days, after which the seedlings began to deteriorate due to nutrient depletion and the limitations of the closed system. Consequently, 18 days old seedlings (4 days old when transferred to the containers + 14 days inside the containers), were harvested, phenotyped, and imaged.

To assess bacterial colonization, samples were taken from both the water agar and roots and inoculated on TSA plates for bacterial counting using CFU methods. Seedlings were air-dried at 60°C and monitored regularly until a constant weight was achieved to determine the dry weight. The dry weight measurements represent the average dry weight of six biological replicates, with each replicate including the entire seedling (both shoots and roots). The dry weight is expressed as mg per seedling. Samples reserved for transcriptomic analysis were immediately frozen in liquid nitrogen and stored at −80°C until further processing.

### 2.3 RNA extraction and quantification

Six seedlings per replicate for each treatment were flash-frozen in liquid nitrogen, and ground into powder, and RNA was extracted using Trizol (Thermo Fisher Scientific, Hemel Hempstead, UK) following the manufacturer’s guidelines. RNA was purified using the QIAGEN RNeasy Mini Elute Kit, and quality was assessed by agarose gel. RNA quantification was determined by analyzing 2 µl on a Qubit fluorometer (Thermo Fisher Scientific, Hemel Hempstead, UK). Samples were diluted with DEPC water and sent to the sequencing service provider (Earlham Institute, Norwich, UK) where Illumina RNA-seq libraries were prepared and sequenced using the HiSeq 2500 platform.

### 2.4 RNAseq processing, quality control, and mapping

Prior to mapping, raw reads were processed using Trimmomatic v.0.33 to remove adapters with the following parameters (optimized after several run tests): ILLUMINACLIP: TruSeq3-PE-2.fa LEADING:15 SLIDINGWINDOW:4:15 MINLEN:30 HEADCROP:12 (Bolger et al., 2014). The quality of the resulting trimmed and cleaned reads was assessed using FastQC v.0.11 (Andrews, 2014). Reads were then mapped to assembly version 3.1 of the *Brachypodium distachyon* genome, downloaded from Phytozome (https://phytozome.jgi.doe.gov). Briefly, reads were mapped to the reference genome using the splice-aware mapper Hisat2 v.2.0.0 (Kim et al., 2015).

### 2.5 Preprocessing and quantification of transcripts

Prior to calling DEGs, preprocessing filtering was performed to remove potential artifacts and assess the quality of the replicates. Count matrices were derived from the bam files above using the Genomic Features and Genomic Alignments R libraries. Transcripts with a count lower than 10 in any samples were discarded. We applied the regularized logarithm transformation (rlog) as implemented in the DESeq2 package to decrease the variance among gene expression values, as proposed by Love et al. (2014). We then calculated a distance matrix between samples and performed a principle component analysis (PCA) to quantify experimental covariates and batch effects among samples and replicates (Van Belle et al., 2004).

### 2.6 Estimating the completeness of transcriptomes

The transcriptome in each sample was assessed for its completeness as a measure of the quality of the sequencing. Clean reads were mapped to the reference genome (above) and were assembled and merged using StringTie v1.1.0 using default parameters (Pertea et al., 2015). The completeness of each transcriptome was assessed using BUSCO on the early-release plantdb set, composed of 1,440 core genes (Simao et al., 2015).

### 2.7 Identification of DEGs using Salmon and DESeq2

Quantification of transcripts was done using Salmon using precomputed mapping files (bam files) computed as described above using the –*ValidateMappings* –*gcBias* and –*numBootstraps* set to 1,000 to improve the quantification (Patro et al., 2017). Derived counts were used as inputs to call DEG using DESeq2 comparing the different treatments to control treatment (sterile water agar). The overlap of DEGs between different treatments was computed using the UpSetR library(doi: https://doi.org/10.1093/bioinformatics/btx364).

### 2.8 Functional annotation of DEGs and GO term enrichment

The reference genome was functionally re-annotated using Blast2GO 5.25 (Pro) as a prior step before computing GO term enrichments (Camacho et al., 2009). The functional annotation was done as follows: BLAST searches were performed on the nr database (release March 2019) using the BLASTx command from ncbi-blast-2.2.28+ release at an *e*-value cut-off of 0.000001 and selecting the top 20 hits (Jones et al., 2014). InterPro searches were performed using InterProScan v.5.18-57 on TIGRFAM, PFAM, SMART, PANTHER, Gene3d, and PIRSF databases (Wu et al., 2004; Letunic et al., 2006; Lees et al., 2010; Haft et al., 2013; Mi et al., 2013; Finn et al., 2016). Finally, enriched GO terms in all three categories: molecular function (MF), biological process (BP), and cellular component (CC) among identified DEGs were identified using the Fisher’s exact test as implemented in Blast2GO 5.25 (Pro) at a *p*-value cut-off of 0.01 (Conesa and Gotz, 2008).

### 2.9 Statistical analysis and visualization

Data based on replicates (at least three biological replicates) were subjected to different statistical analysis methods using several packages. Analysis of variance (ANOVA) test to determine the significance between the different treatments was carried out using Costas (CoHort software, Pacific Grove, CA, USA). The heat map was generated based on using Pearson and Ward for distance measure and clustering algorithm using the XLSTAT package. Analysis using the MapMan package was used to visualize differences in biological functions among different treatments (Usadel et al., 2009).

## 3 Results

### 3.1 *B. velezensis* CBE-mediated osmotic stress tolerance in *B. distachyon*

**Figure 1:**
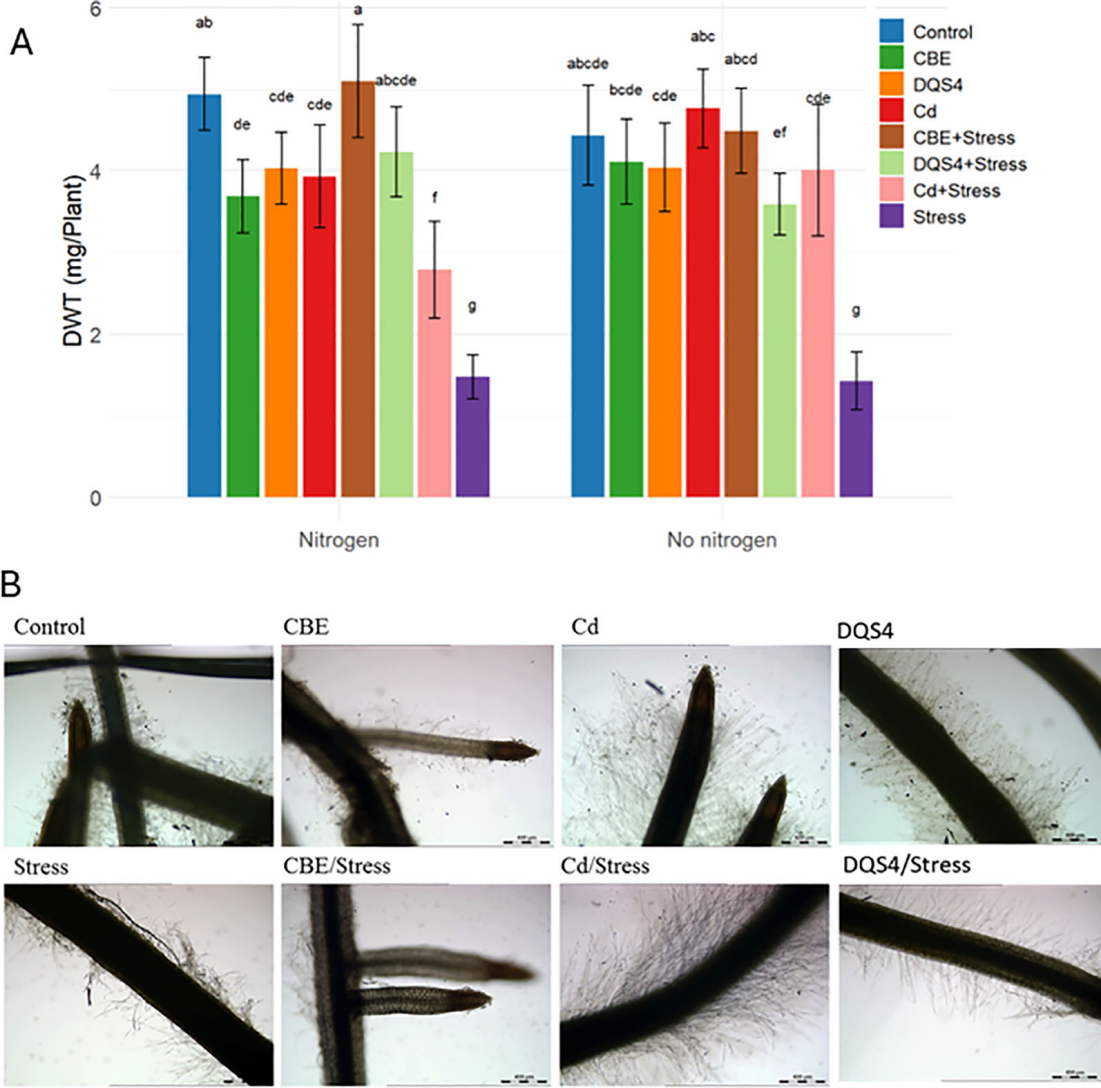
Response of *B*. *distachyon* bd21 seedlings to the inoculation with novel halotolerant endophytic bacterial strain *B*. *velezensis* CBE, *A. olearius* DQS4, and *A. brasilense* Cd (reference control strains). Four-day-old seedlings were grown for 14 days either in sterile water agar (2.5 g/ml) or in 5% PEG8000 (osmotic stress) under normal nitrogen supply (3 mM NH_4_NO_3_) or no nitrogen supply. **(A)** Seedling dry weight (mg/plant). Bars indicate standard deviation (*n* = 6). Treatments labeled with identical small letters are not significant at *p* ≤ 0.01. **(B)** Photomicrographs showing root phenotypes.

The effect of osmotic stress (5% PEG8000) on *B. distachyon* growth was observed after 18 days (postgermination). As illustrated in **Figure 1**, the stressed seedlings exhibited a clear deleterious impact. The stress treatment significantly reduced the seedlings’ dry weight and resulted in a characteristic stressed root phenotype (**Figures 1A, B**).

Seedlings treated with *B. velezensis* CBE showed far greater tolerance to the osmotic stress. Bacterial treatment had a significant effect on osmotic stressed *B. distachyon*. Hence, the inhibitory effects of PEG treatment on the plant phenotypes were halted after CBE treatment, where a significant dry weight increase was evident in stressed CBE-treated seedlings (**Figure 1A**). CBE also resulted in different root phenotypes than unstressed and stressed *B. distachyon* seedlings (**Figure 1B**).

The ability of *B. velezensis* CBE to mediate osmotic stress tolerance in *B. distachyon* was compared with the relatively similar stimulation effect of the well-known PGPB strains *A. brasilense* Cd and *A. olearius* DQS4 under limited and normal nitrogen regimes. We did not find a clear difference in *B. distachyon* growth after treatment with any of the strains under stress conditions, as all strains appeared to effectively improve *B. distachyon’s* ability to withstand osmotic stress imposed by PEG treatment (**Figure 1**). However, *B. velezensis* CBE was superior under normal nitrogen conditions, as a significantly higher dry weight was recorded in stressed *B. velezensis* CBE-treated seedlings compared with stressed *A. brasilense* Cd and *A. olearius* DQS4-treated seedlings grown in nitrogen-supplemented water agar. In the case of seedlings grown without nitrogen, both strains significantly increased *B. distachyon* dry weight under stress conditions (**Figure 1A**). *A. brasilense* Cd treatment-induced lateral roots and root hair growth in both stressed and stressed seedlings, which was not the case in roots of *B. velezensis* CBE-treated seedlings (**Figure 1B**).

Bacterial colonization was assessed in the same sterile system by quantifying bacterial presence using the CFU method on TSA plates. A significant increase in the total bacterial count for *B. velezensis* CBE, *A. brasilense* Cd, and *A. olearius* DQS4 was observed in the medium, roots, and surface-disinfected roots under all tested conditions, compared to the uninoculated treatment. Both *B. velezensis* CBE and *A. olearius* DQS4 demonstrated an equal ability to colonize plant roots endophytically, as indicated by the CFU counts. However, in plants treated with the *A. brasilense* Cd strain, no significant increase in bacterial count was observed in the disinfected roots, suggesting the nonendophytic nature of this strain (**Supplementary Figure S2**).

### 3.2 Profiling of the *B. distachyon* seedling transcriptome

Transcriptome profiling was conducted on 18-day-old *B. distachyon* seedlings grown under osmotic stress (5% PEG8000) and/or nitrogen supply (3 mM ammonium nitrate) to identify differentially expressed genes (DEGs) in response to treatment with three different PGPB strains (*B. velezensis* CBE, *A. brasilense* Cd and *A. olearius* DQS4) using shotgun RNA-Seq analysis. The transcriptomes of all treatments were compared to the transcriptome obtained for the control treatment (14-day-old seedlings grown in liquid water agar). Data obtained for all treatments were further analyzed for hierarchical clustering, and a heat map was generated to illustrate global transcriptional changes (**Supplementary Figure S3**). Data presented in **Table 1** shows the numbers of significant identified DEGs (*p*-values < 0.01 and log_2_ fold-change > 2). Nitrogen treatment had the most pronounced transcriptional effects on *B. distachyon* seedlings, where 8,584 DEGs were counted (4,144 upregulated and 4,440 downregulated DEGs). Osmotic stress (5% PEG8000) treatment, on the other hand, resulted in much lower transcriptional changes in *B. distachyon* seedlings (160 DEGs, 78 upregulated and 82 downregulated).

**Table 1:** Numbers of significant (*p* < 0.01, *n* = 3) differentially expressed genes (DEGs) detected in *B. distachyon* Bd2.

Treatments	Differentially expressed genes (DEGs)		
	Upregulated	Downregulated	Total
Stress	78	82	160
Nitrogen	4,144	4,440	8,584
Stress + nitrogen	2,750	2,909	5,659
*B. velezensis* CBE	50	281	331
*A. brasilense* Cd	322	105	427
*A. olearius* DQS4	127	221	348
Stress + CBE	52	21	73
Stress + Cd	601	477	1,078
Stress + DQS4	1,045	970	2,015
Nitrogen + CBE	1	17	18
Nitrogen + Cd	192	250	442
Nitrogen + DQS4	81	119	200
Stress + nitrogen + CBE	24	38	62
Stress + nitrogen + Cd	653	1,181	1,839
Stress + nitrogen + DQS4	572	883	1,455

Treatments represent seedlings inoculated with novel halotolerant endophytes (*B. velezensis* CBE), and control strains (*A. brasilense* Cd or *A. olearius* DQS4). Four-day-old seedlings were grown for 14 days either in sterile water agar (2.5 g/ml) or in 5% PEG8000.

All tested PGPB strains were able to modulate gene expression in *B. distachyon* (**Table 1**). Transcriptional changes in response to each tested PGPB strain were diverse and depended on the bacterial strain and the stress or nitrogen conditions. Compared to the other tested PGPB strains (*A. brasilense* Cd and *A. olearius* DQS4), *B. velezensis* CBE treatment-induced minimal transcriptional modulations in *B. distachyon* seedlings under all tested conditions. The maximum DEGs recorded in CBE-treated seedlings were found in unstressed *B. distachyon* grown under nitrogen-free conditions (331 DEGs, 50 upregulated and 281 downregulated) (**Supplementary Table S2**). Only 73 DEGs were identified in *B. velezensis* CBE-treated seedlings under stress conditions. DGEs number further fell to 18 in seedlings grown under normal nitrogen conditions.

### 3.3 Overlapping DEGs in PGPB-treated *B. distachyon*

**Figure 2:**
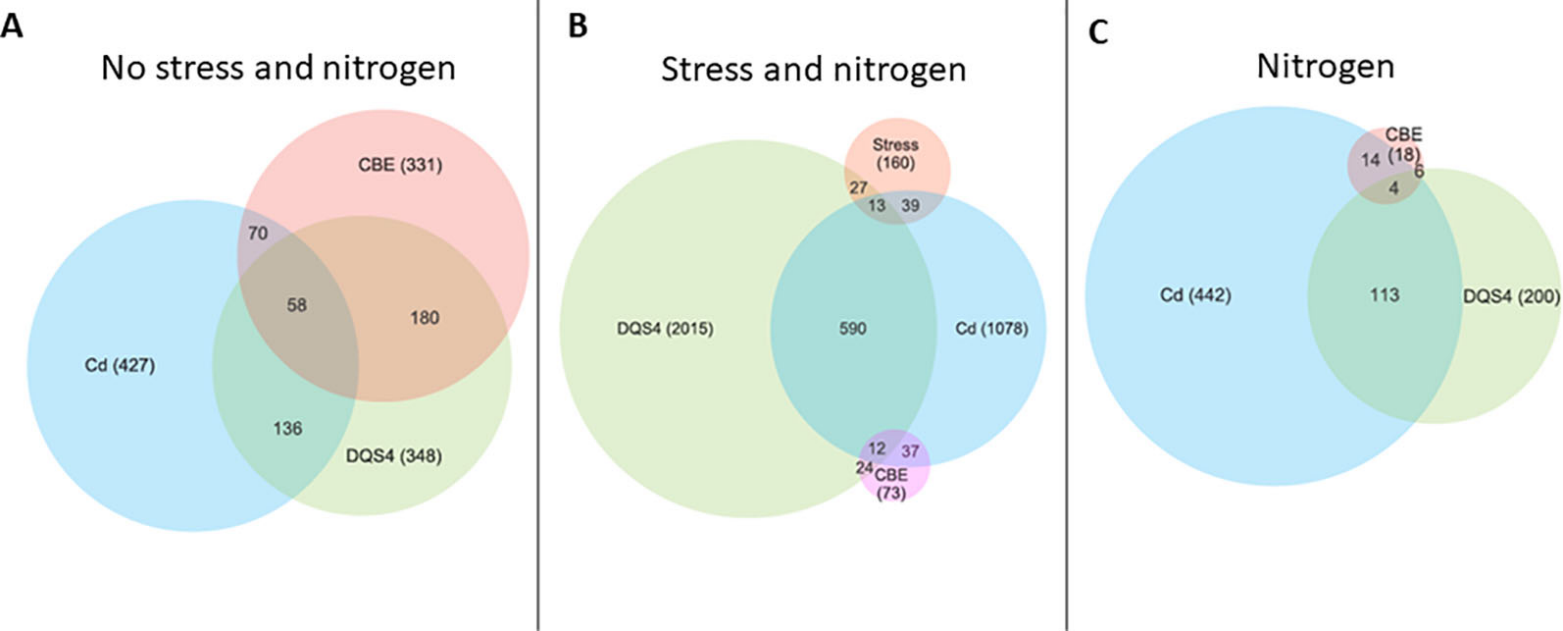
Venn diagrams showing the numbers of unique and overlapping differentially expressed genes (DEGs; significant difference at *p* ≤ 0.01, *n* = 3) in 14-day-old *B*. *distachyon* Bd2 seedlings treated with a novel halotolerant endophyte (*B. velezensis* CBE) and control strains (*A. brasilense* Cd or *A. olearius* DQS4) compared to control (not treated with bacteria). **(A)** Unstressed seedlings. **(B)** Osmotic stressed (5% PEG8000) seedlings. **(C)** Normal nitrogen supply (3mM NH_4_NO_3_).

More overlapping DEGs between all tested PGPB strains were detected in unstressed *B. distachyon.* Data illustrated in **Figure 2A** show 180 overlapping DEGs between *B. velezensis* CBE and *A. olearius* DQS4 and 58 overlapping DEGEs between *B. velezensis* CBE and *A. brasilense* Cd. In stressed seedlings, it was evident that Cd and DQS4 have resulted in several transcriptional modifications. Furthermore, 590 DEGs were overlapped between *A. brasilense* Cd and *A. olearius* DQS4 (**Figure 2B**). Very few overlapping DEGs were found in *B. velezensis* CBE-treated stressed seedlings; hence, only 37 overlapping DEGs were identified between CBE and *A. olearius* DQS4, while 24 overlapping DEGs were detected between *A. brasilense* Cd and *B. velezensis* CBE (**Figure 2B**). It was noticeable that, in contrast to the 113 overlapping DEGs between *A. brasilense* Cd- and *A. olearius* DQS4-treated seedlings, there were only six overlapping DEGs between *B. velezensis* CBE and *A. olearius* DQS4, and 14 overlapping DEGs between *B. velezensis* CBE and *A. brasilense* Cd (**Figure 2C**).

### 3.4 GO enrichment analysis showed potential biological processes associated with different PGPB treatments in unstressed *B. distachyon* seedlings

**Figure 3:**
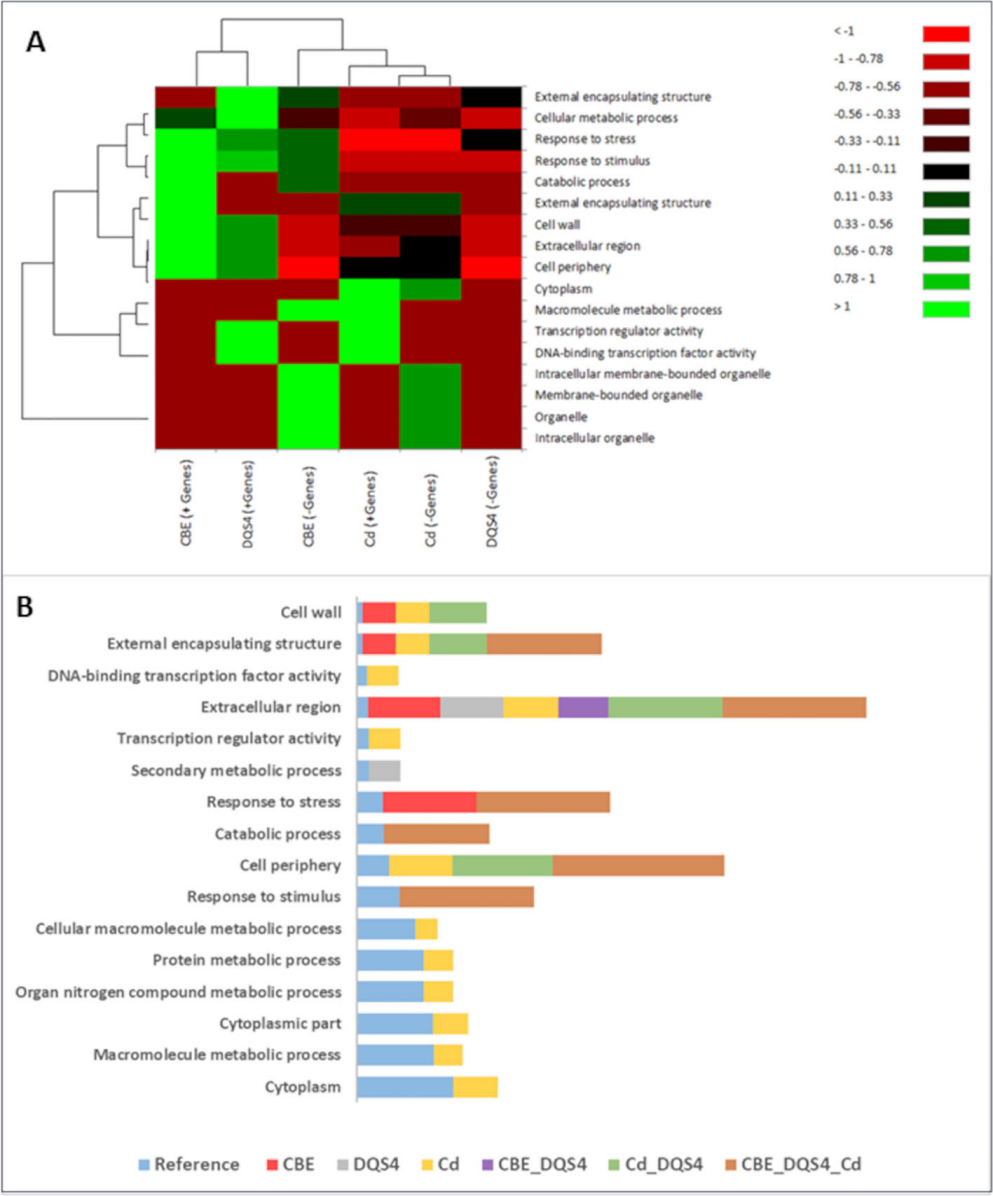
Gene Ontology annotations (GO) for significant DEGs found in 18-day-old *B. distachyon* Bd2 seedlings treated with a novel halotolerant endophyte (*B. velezensis* CBE) and control strains (*A. brasilense* Cd or *A. olearius* DQS4) compared to control (not treated with bacteria). Annotations were performed for the three sub-trees of GOs: biological process, molecular function, and cellular component, using Blast2GO tools. **(A)** Heat map showing variation in enriched GOs between up- and downregulated DEGs in *B*. *distachyon* Bd2 seedlings following bacterial treatments. The map was generated based on Pearson and Ward methods for distance measurement and clustering with the XLSTAT package. **(B)** Enrichment bar chart showing over- and underrepresented enriched functions along with the corresponding sequence percentages for unique and overlapping DEGs in *B*. *distachyon* Bd2 seedlings after bacterial treatments. Sequence % compare treated plants with the reference *B*. *distachyon* Bd2 genome. A higher sequence % for a given function (compared to the reference) indicates that the function is overrepresented in the treated plant.

To identify potential biological functions of the DEGs (up- and downregulated) by each PGPB strain treatment, we performed a GO terms enrichment analysis of those genes in three sub-trees of GOs (biological process, molecular function, and cellular component). The enrichment analysis showed variable GO representation in unstressed *B. distachyon* seedlings in response to treatment with each PGPB strain. The heatmap illustrated in **Figure 3A** shows that upregulated genes due to treatment with *B. velezensis* CBE and *A. olearius* DQS4 strains enriched relatively similar GOs that involved several biological functions, including responses to stresses and stimulus and cellular metabolic processes. There was a clear contrast in the GOs enhanced for the downregulated genes in response to *B. velezensis* CBE and *A. olearius* DQS4, as they were clearly separated in the heatmap (**Figure 3A**). Molecular functions related to cellular membranes and macromolecule metabolic processes represented GOs related to the downregulated genes in *B. velezensis* CBE-treated seedlings (**Figure 3A**). Unlike *B. velezensis* CBE and *A. olearius* DQS4, up- and downregulated genes in *A. brasilense* Cd-treated seedlings resulted in related GO enrichment, as illustrated in the clustering analysis. However, it was clear that some GOs were enriched for the upregulated genes, such as cytoplasmic and transcriptional regulation functions, while others were overrepresented for the downregulated genes, such as organelle-related functions (**Figure 3A**).

GO term enrichment analysis for DEGs unique to *B. velezensis* CBE treatment showed that only four functions were overrepresented (**Figure 3B**). Cell wall, external encapsulating structure, and extracellular region functions were all found to be overrepresented in response to all tested bacterial strains (**Figure 3B**). GO term enrichment analysis for overlapping DEGs in response to the three tested bacterial strains showed enriched six biological functions including response to stresses and stimulus and cell periphery (**Figure 3B**).

### 3.5 GO enrichment analysis of DEGs showed potential biological processes associated with different DQS4 and Cd treatments, but not CBE treatment, in *B. distachyon* seedlings under osmotic stress and normal nitrogen supply conditions

**Figure 4:**
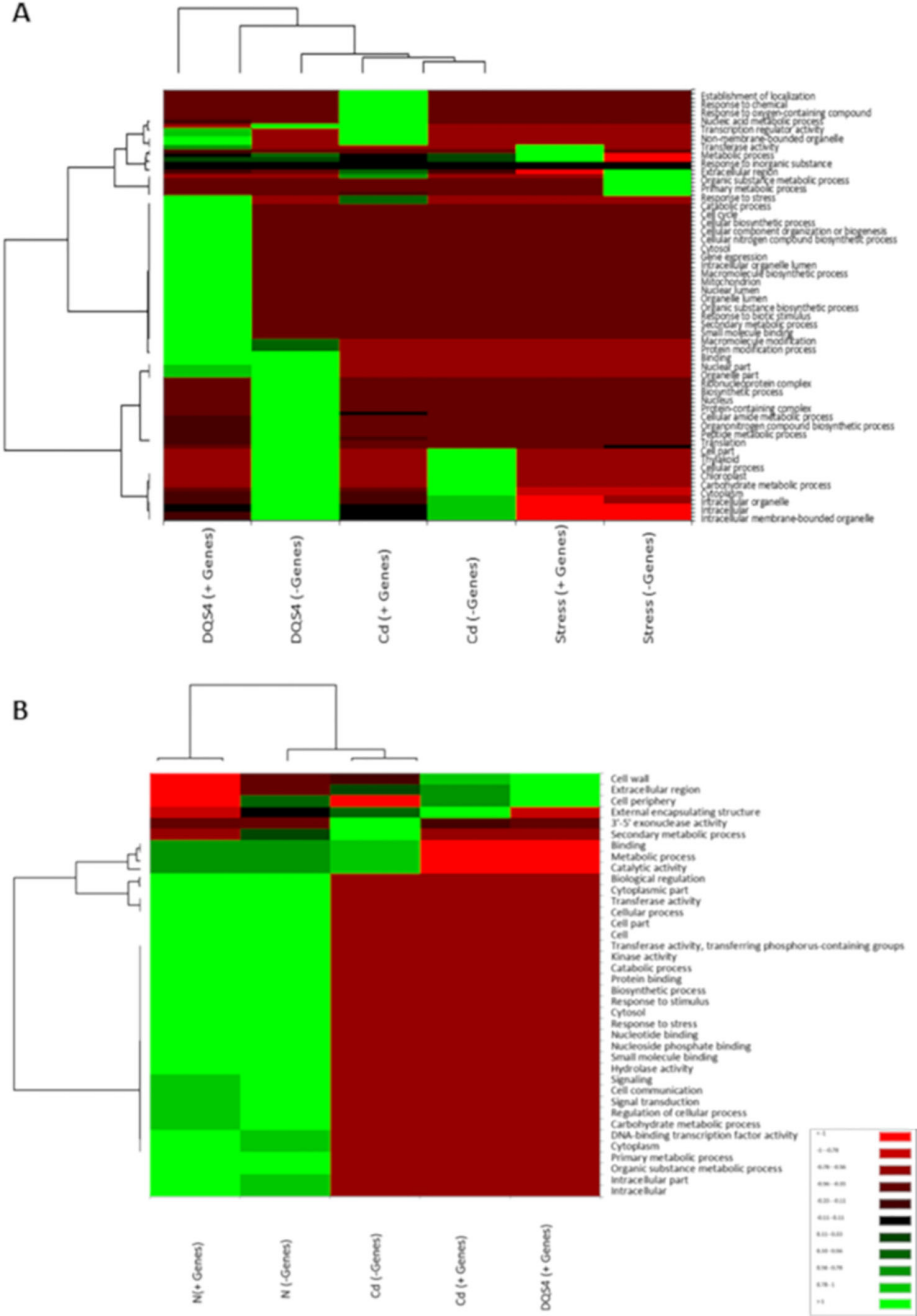
Gene Ontology annotations (GO) for significant DEGs found in 18-day-old stressed *B*. *distachyon* Bd2 seedlings treated with bacterial strains (*A. brasilense* Cd or *A*. *olearius* DQS4) compared to control (not treated with bacteria). The annotations were performed for the three sub-trees of GOs: biological process, molecular function, and cellular component, using Blast2GO tools. **(A)** Heat map depicting variations in enriched GOs between up- and- downregulated DEGs in osmotically stressed (5%PEG8000) *B*. *distachyon* Bd2 seedlings after bacterial treatments. **(B)** Heat map showing variation in the enriched GOs between up- and downregulated DEGs in *B*. *distachyon* Bd2 seedlings grown under normal nitrogen supply (3 mM NH_4_NO_3_). The maps were generated using Pearson and Ward methods for distance measurement and clustering, implemented in the XLSTAT package.

Up- and downregulated DEGs identified in *B. distachyon* Bd21 seedlings treated with sterile TSB media (control), *B. velezensis* CBE, DQS4, or Cd bacterial strains in response to either osmotic stress (5% PEG8000) or nitrogen supply (3 mM NH_4_NO_3_) were subjected to GO term enrichment analysis across the three sub-trees of GOs (biological process, molecular function, and cellular component). Osmotic stress treatment led to the enrichment of GOs often associated with stress response, such as primary and organic substance metabolic process (**Figure 4A**). Nitrogen treatment induced substantial transcriptomics changes; therefore, several functions were overrepresented in *B. distachyon* Bd21 seedlings grown under nitrogen supply (**Figure 4B**). Functional enrichment for DEGs in response to bacterial treatments under osmotic stress or nitrogen supply conditions was highly variable and strain-dependent. However, the most noticed finding was that the GOs enrichment for *B. velezensis* CBE-related DEGs did not indicate any potential biological functions in seedlings grown under either osmotic stress or nitrogen supply conditions. On the other hand, contrasting functional enrichments were found for DEGs identified in DQS4- or *A. brasilense* Cd-treated seedlings grown under either osmotic stress or nitrogen supply conditions (**Figures 4A, B**).

**Figure 5:**
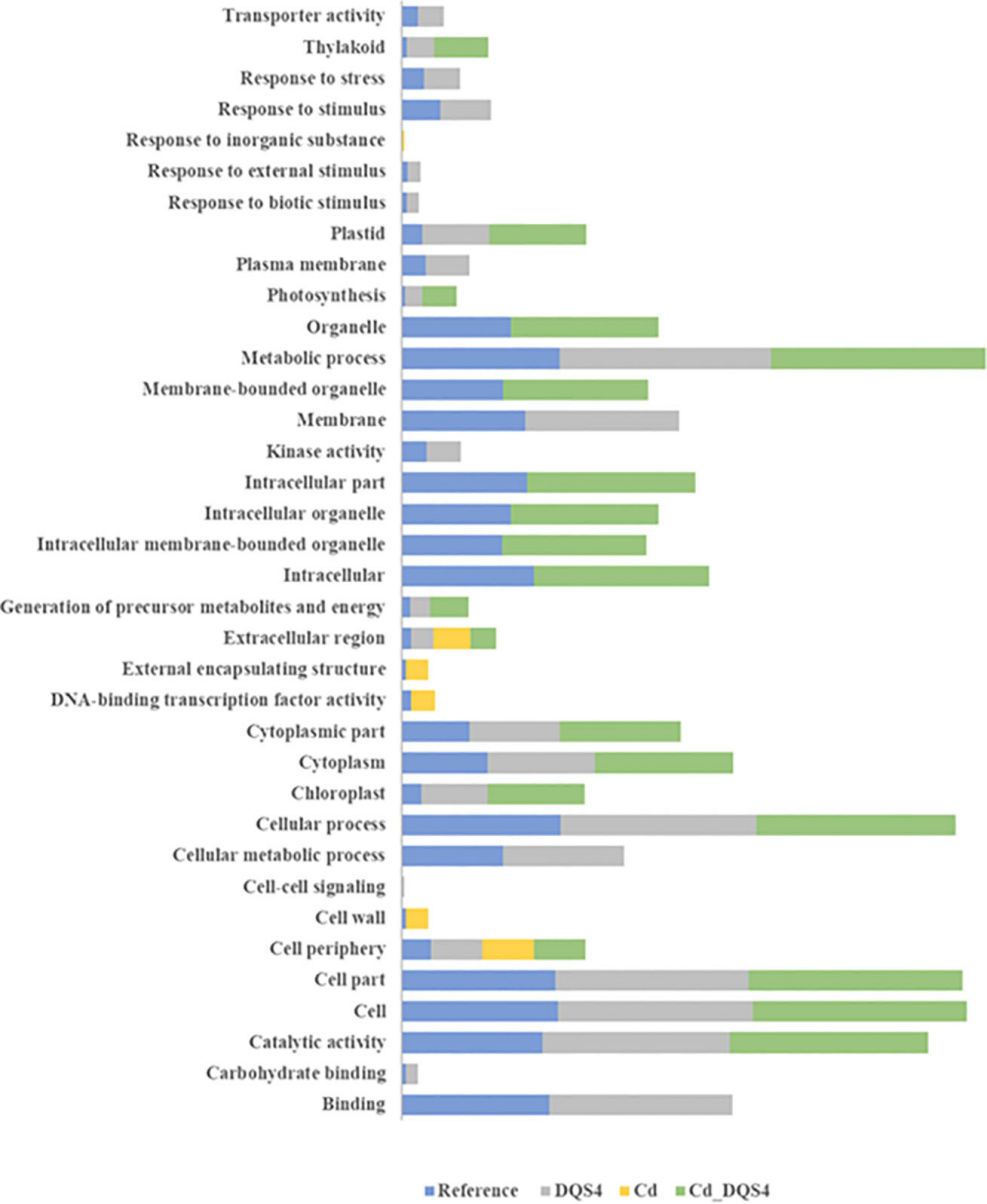
Enrichment bar chart showing over- and underrepresented enriched functions and the corresponding sequence percentages for unique and overlapping DEGs in osmotically stressed (5%PEG8000) *B*. *distachyon* Bd2 seedlings after bacterial treatments. The sequence % represent comparisons between treated plants are compared with sequence % in reference *B*. *distachyon* Bd2 genome. Higher sequence % for a given function (compared to the reference) indicate that the function is overrepresented in the treated plant.

Functional enrichment analysis of unique DEGs resulting from *A. olearius* DQS4 treatment revealed that several GO terms were overrepresented in *B. distachyon* Bd21 seedlings grown under osmotic stress conditions (**Figure 5**). Furthermore, most of these functions were enriched in DEGs that overlapped between *A. olearius* DQS4 and *A. brasilense* Cd treatments (**Figure 5**).

### 3.6 Minor transcriptome modifications in *B. distachyon* Bd21 during *B. velezensis* CBE-mediated osmotic stress tolerance

**Figure 6:**
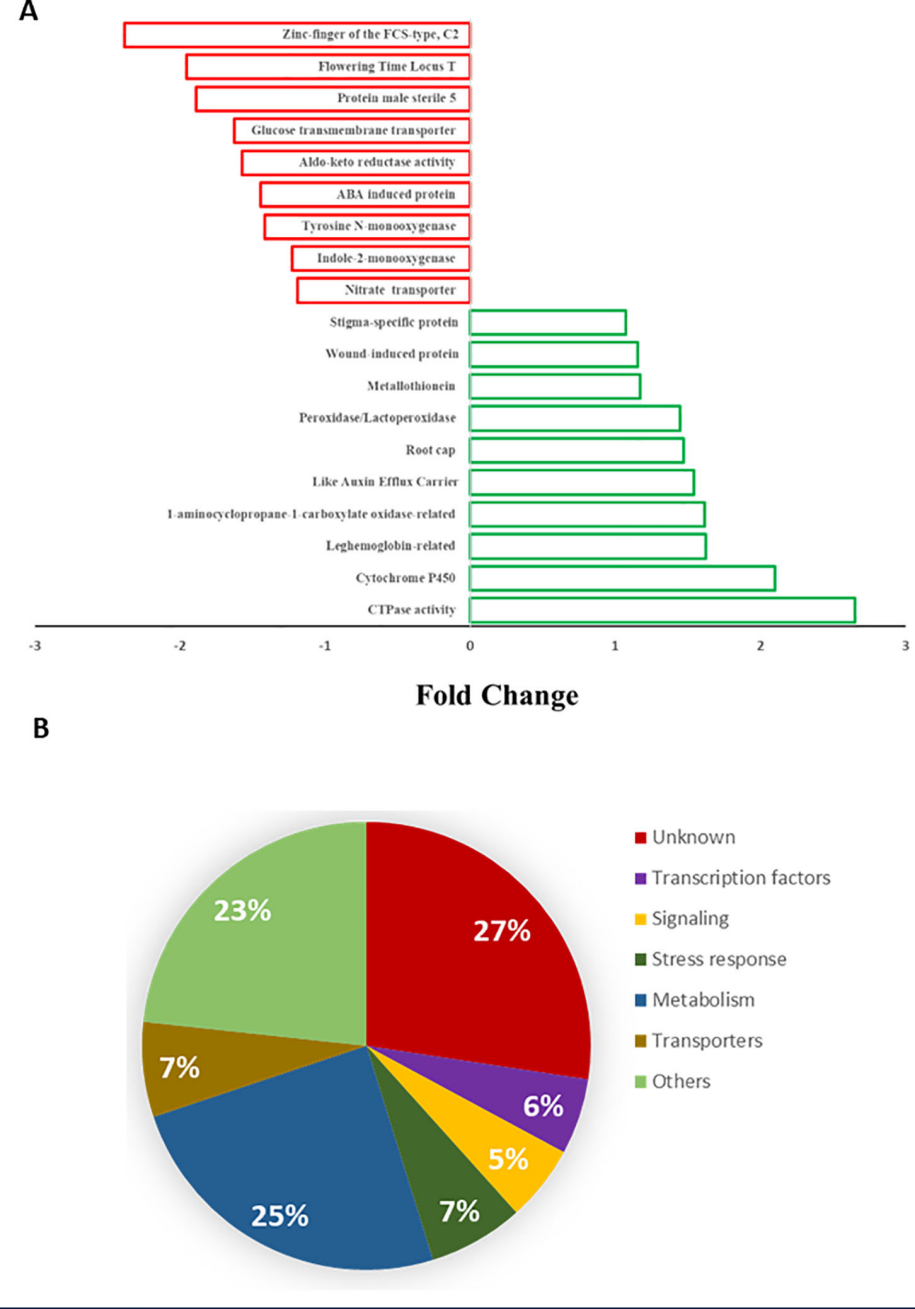
Representing DEGs detected in *B*. *distachyon* Bd2 during *B*. *velezensis* CBE-mediated osmotic stress tolerance. **(A)** Fold change (relative to control unstressed treatment). **(B)** Putative comparative classification according to the biological function of genes corresponding to the DEGs based on database queries using BLAST.

The effect of *B. velezensis* CBE treatment on the overall *B. distachyon* Bd21 transcriptome was relatively minor. Hence, only 73 genes were found to show significant differential expressions (the data illustrated in **Figure 6A** shows fold changes in the expression of genes representing up- and downregulated genes). Functional classification of the identified genes showed that up to 27% did not correspond to any known genes (**Figure 6B**). Most of the functionally known genes (25%) were involved in plant metabolism (**Figure 6B**). The remaining genes were assigned to different functions, including signaling, cellular transport, and transcriptional regulation (**Figure 6B**).

**Figure 7:**
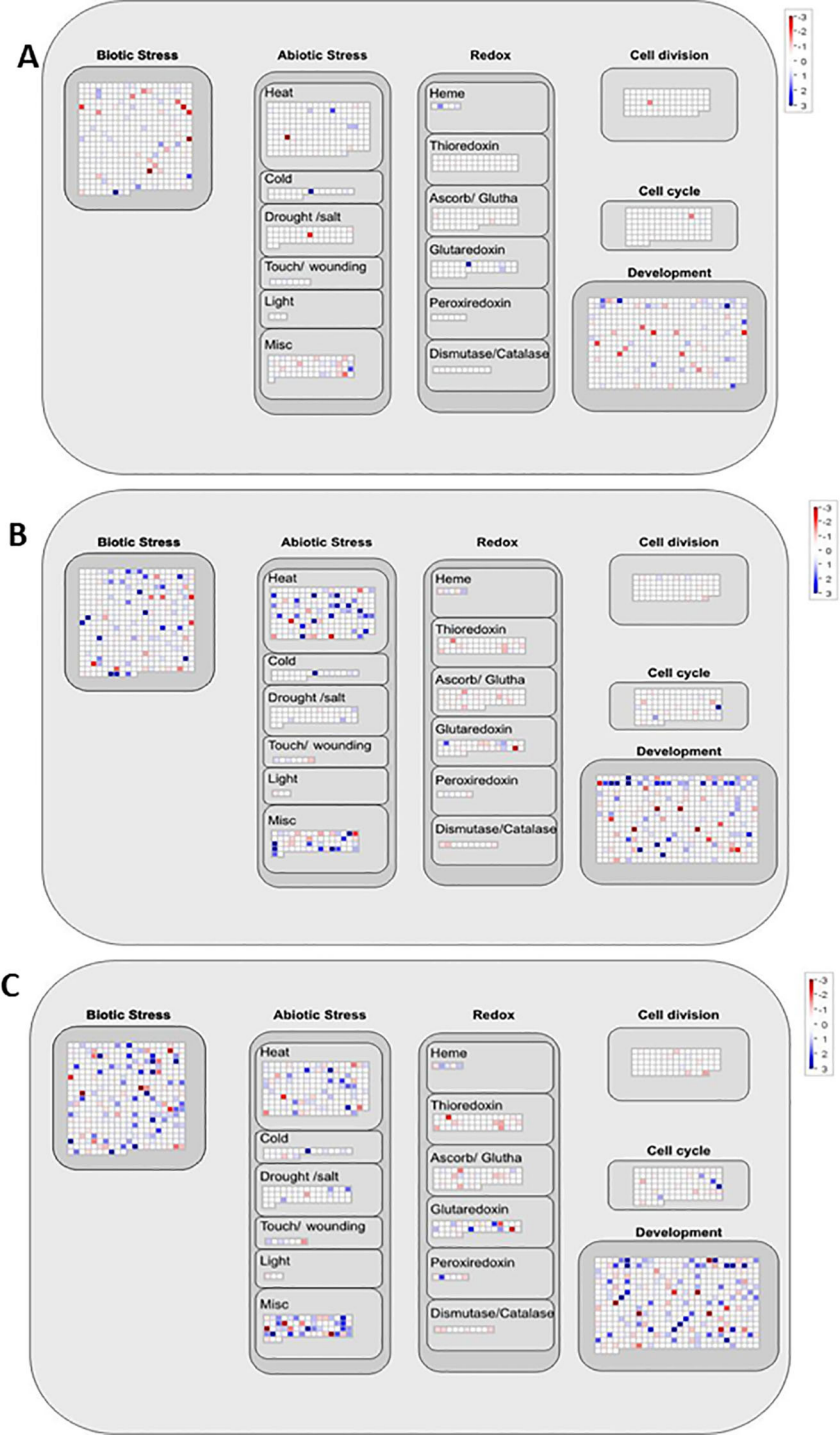
MapMan overview of transcriptional changes involved in overall cellular responses in *B*. *distachyon* Bd21 stressed seedlings treated with CBE **(A)**, DQS4 **(B)**, and Cd **(C)**. Each square represents a separate DEG; red indicates that gene expression was induced, and blue indicates that gene expression was repressed compared with the control.

The transcriptomes of *B. velezensis* CBE, *A. olearius* DQS4, and *A. brasilense* Cd-treated stressed (5% PEG8000) *B. distachyon* Bd21 seedlings were mapped to different functional categories using the MAPMan tool to reveal molecular processes associated with each strain’s ability to mediate stress tolerance. The cellular response overview confirmed that all tested strains impacted genes involved in various cellular responses, including redox, biotic, and abiotic stress responses. However, it was clear that *B. velezensis* CBE had a lower influence on the overall cellular response than DQS4 and *A. brasilense* Cd (**Figure 7**). Developmental and biotic stress-responsive genes were the most represented genes in the transcriptome of *B. velezensis* CBE-treated seedlings under osmotic stress (**Figure 7A**). That was also the case in *A. olearius* DQS4 and *A. brasilense* Cd-treated seedlings, yet significantly more genes were involved in mounting the required developmental and biotic stress responses (**Figures 7B, C**). Furthermore, several abiotic stress-related genes were found in *A. olearius* DQS4 and *A. brasilense* Cd-treated seedlings under osmotic stress conditions but not in the *B. velezensis* CBE-treated seedlings, where only a few genes were associated with abiotic stress response (**Figure 7**).

**Figure 8:**
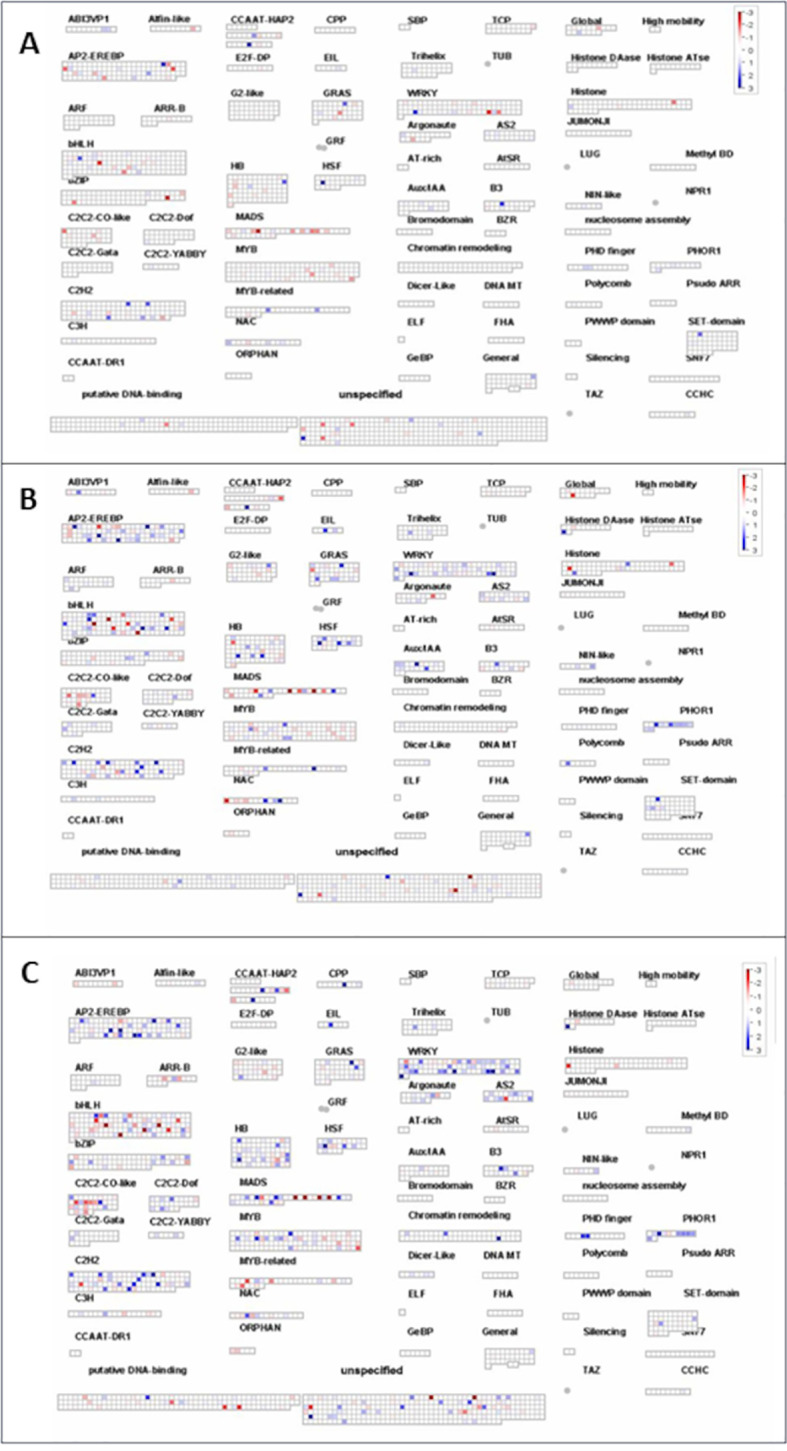
MapMan overview of transcription factors expression changes in *B*. *distachyon* Bd21 stressed seedling treated with CBE **(A)**, DQS4 **(B)**, and Cd **(C)**. Each square represents a separate DEG; red indicates that gene expression was induced, and blue indicates that gene expression was repressed compared with the control.

MAPMan was also used to map transcription factors contributing to the strain’s potential to mediate stress tolerance. It was evident that *B. velezensis* CBE treatment has resulted in minor changes in the expression of different transcription factors compared to the other two tested strains (**Figure 8**). For instance, upregulation of one AP2-EREBP transcription factor was detected in seedlings treated with *B. velezensis* CBE under stress conditions (**Figure 8A**). However, more than five AP2-EREBP transcription factors were upregulated in *A. olearius* DQS4-treated seedlings (**Figure 8B**), and more than nine were upregulated in *A. brasilense* Cd-treated seedlings (**Figure 8C**). A similar trend was observed with other transcription factors, such as BHL-Hs, WARYs, and MYBs (**Figure 8**).

## 4 Discussion

The use of microorganisms to enhance plant tolerance to abiotic stress is considered an environmentally sustainable strategy for mitigating stress-related effects. Consequently, extensive research is underway to identify and characterize novel microorganisms with potential to augment abiotic stress tolerance (Singh et al., 2023). In this study, we demonstrate the ability of the newly discovered halotolerant strain, *B. velezensis* CBE, to enhance osmotic stress tolerance in *B. distachyon* Bd2 seedlings under both normal and nitrogen-depleted conditions. Notably, the effectiveness of *B. velezensis* CBE in improving osmotic stress tolerance was comparable to or even superior (especially under normal nitrogen levels) to the well-established PGPB species (*A. brasilense* Cd).

Noticeably, while the application of *B. velezensis* CBE did not stimulate the formation of lateral roots and root hairs in treated plants, it is crucial to note that Cd treatment clearly promoted these features. The influence of various beneficial microbes, including *Azospirillum*, on plant root systems is well-documented in scientific literature, as reviewed by Grover et al. (2021). This effect is often attributed to the microbes’ ability to enhance plant nutrient and water uptake (Grover et al., 2021). Additionally, changes in the root system response to beneficial microbes may be attributed to changes in signaling within key hormonal pathways that regulate plant root development, such as auxin and cytokinin (Verbon and Liberman, 2016). However, the limited impact on the root systems of plants treated with *B. velezensis* CBE suggests that each bacterial species may employ a unique mechanism to confer advantageous traits to plants under stressful conditions. The differences in observed root phenotypes among the tested strains may be attributable to the significant phylogenetic divergence between species. Furthermore, the distinct ecological niches occupied by each respective strain—*B. velezensis* CBE as a plant endophyte and *A. brasilense* Cd as a plant rhizosphere colonizer—could exert varying effects on the host plant. Consequently, these disparities in microbial characteristics are expected to lead to diverse responses. These variations could lead to the production of different microbial bioactive metabolites, including phytohormones, osmolytes, and antioxidants, which may directly influence the plant host in distinct ways.

Considering the distinct phenotypic responses observed in *B. velezensis* CBE-treated plants, we hypothesized that the plant transcriptional regulation involved in mediating osmotic stress tolerance in *B. distachyon* Bd2 differs from that induced by other beneficial microbes, such as *A. brasilense* Cd. To investigate this hypothesis, we conducted comprehensive transcriptomic profiling on *B. distachyon* Bd2 seedlings treated with *B. velezensis* CBE, aiming to identify the molecular factors contributing to the induced osmotic stress tolerance. Additionally, we performed transcriptomic analysis on plants treated with other beneficial microbes, specifically *A. brasilense* Cd and *A. olearius* DQS4, to elucidate similarities and differences in plant transcriptional responses to each bacterial strain.

Beneficial microbes possess the capacity to reprogram the plant’s transcriptomes, frequently leading to shifts in gene expression profiles (Mukherjee, 2022). These modifications can subsequently govern various molecular and metabolic processes (Mukherjee, 2022). In the present study, we observed transcriptional changes in *B. distachyon* Bd21 seedlings treated with the novel *B. velezensis* CBE strain, both under normal and osmotic stress conditions. As anticipated, substantial alterations in the transcriptome were also noted in seedlings treated with *A. brasilense* Cd and *A. olearius* DQS4. However, the extent of the plant transcriptional modulations varied significantly among the different strains, as indicated by the substantially higher numbers of DEGs identified in the *A. brasilense* Cd and *A. olearius* DQS4-treated plants under all tested conditions. Furthermore, it appeared that *A. brasilense* Cd and *A. olearius* DQS4 induced more pronounced changes in the plant transcriptome profiles under both normal and osmotic stress conditions compared to *B. velezensis* CBE, indicated by the higher DEGs in seedlings treated with *A. brasilense* Cd and *A. olearius* DQS4.

Numerous genes have been implicated in modulating the interactions between plants and beneficial microbes (Liu et al., 2023). Our results show that treatment with strains *B. velezensis* CBE, *A. olearius* DQS4, and *A. brasilense* Cd-induced notable changes in plant gene expression profiles, observable under both standard growth conditions and in response to osmotic stress. The genes affected are associated with a diverse array of plant molecular and cellular functions, including but not limited to heat shock proteins (HSPs), cellular transport mechanisms, metal-ion binding proteins, components of signal transduction pathways, and transcription factors. Such molecular processes are posited to be essential for the facilitation of abiotic stress resilience by the beneficial microbes (Conde et al., 2011; Scieglinska et al., 2019; Mukherjee, 2022; Liu et al., 2023). Consequently, it was evident that all microbial treatments were capable of mounting the requisite molecular alterations. Notably, transcriptional analysis revealed that each strain modulated a distinct set of genes to influence relatively comparable biological processes. However, strain *B. velezensis* CBE was observed to effect similar functional outcomes by regulating a smaller subset of genes, thereby suggesting a more targeted or efficient molecular regulation, in contrast to the broader gene expression changes induced by strains *A. olearius* DQS4 and *A. brasilense* Cd.

Transcription factors (TFs) play a key role in regulating various cellular processes, including plant responses to stresses and interactions with microbes (Strader et al., 2022). Among these, the MYB family of proteins, present in all eukaryotes, is known for its diversity and functionality. These proteins typically function as transcription factors, possessing varying numbers of MYB domain repeats that enable them to bind to DNA (Yang et al., 2021). We observed differential expression of several *MYB* genes in plants treated with all tested beneficial microbes. Previous studies have highlighted the involvement of *MYBs* in plant responses to diverse abiotic and biotic stimuli, including stress factors, plant pathogens, and beneficial microbes (Sarosh et al., 2009; Ambawat et al., 2013; Yang et al., 2021; Biswas et al., 2023). For example, *MYB102* and *MYB41* have been linked to resistance against insects, as well as wounding and osmotic stress tolerance in Arabidopsis (Zhu et al., 2018). Additionally, *MYB41* was found to regulate ABA-mediated stomatal closure in response to abiotic stresses (Wei et al., 2020).

WRKY transcription factors play a crucial role in regulating the complex network of signaling pathways in plants in response to biotic and abiotic stresses, as well as interactions with beneficial microbes. For instance, several WRKYs were found to be associated with improved drought stress tolerance in several plants (Li et al., 2024). Upregulation of certain WRKY transcription factors appears to be crucial for microbes to mediate abiotic stress tolerance in plants, as suggested by Abd El-Daim et al. (2018), who reported that the *B. velezensis* 5113 lost its ability to mediate drought and heat stress tolerance after knocking down ABA-responsive WRKYs, indicating the involvement of ABA signaling in microbially mediated abiotic stress tolerance. Our results show both up- and downregulation of several *WRKYs* in plants treated with the beneficial microbes *B. velezensis* CBE, *A. olearius* DQS4, and *A. brasilense* Cd. It is also evident that the number of *WRKYs* exhibiting differential expression varies depending on the strain. For instance, plants treated with *B. velezensis* CBE showed differential expression in only three *WRKY* genes.

## 5 Conclusions

Microbially mediated abiotic stress tolerance in plants is an important process, long regarded as an eco-friendly approach to sustainable agriculture. This study demonstrates the potential of the novel halotolerant endophyte *B. velezensis* CBE in enhancing osmotic stress tolerance in *B. distachyon* Bd21 seedlings. We further explored the molecular modulations in plants associated with *B. velezensis* CBE activity through transcriptome profiling, which revealed that *B. velezensis* CBE regulates several molecular functions known to be involved in microbially induced stress tolerance. By comparing *B. velezensis* CBE with other microbes capable of mediating stress tolerance in plants, we highlighted that molecular regulation was strain-dependent. The critical difference among the microbes was the cellular and molecular reprogramming required by *B. velezensis* CBE to confer osmotic stress tolerance in *B. distachyon* Bd21 seedlings. This was achieved through subtle changes in gene expression that regulate these functions. For instance, in plants treated with *A. brasilense* Cd and *A. olearius* DQS4, we observed both up- and downregulation of numerous transcription factors, such as *MYBs* and *WRKYs*. In contrast, *B. velezensis* CBE-inoculated plants exhibited differential expression in only a few of these transcription factors, suggesting that *B. velezensis* CBE’s impact on plant transcription is more specific compared to the broader effects observed with the other tested microbes.

This work is significant because it highlights how different PGPB strains can mediate stress tolerance through relatively similar molecular processes, yet each strain induces these processes through distinct transcriptomic changes. Understanding these differences is crucial and may underpin the design of microbial synthetic communities comprising multiple strains. It will be particularly interesting to investigate plant responses to combinations of PGPB and to determine to what extent these responses are predicted by the response to individual strains. It can be expected that designing a consortium of strains with complementary gene expression changes may enhance stress resilience compared to single strains.

## Data Availability

The data for this study have been deposited in the European Nucleotide Archive (ENA) at EMBL-EBI under accession number PRJEB36975. It includes the fastq files with the filtered reads and bam files with the mapped reads.
