# Supplementary material for: Halotolerant bacterial endophyte Bacillus velezensis CBE mediates abiotic stress tolerance with minimal transcriptional modifications in Brachypodium distachyon
Source: Front Plant Sci. 2025 Jan 10;15:1485391. doi: 10.3389/fpls.2024.1485391 (PMC11757260; doi:10.3389/fpls.2024.1485391)

Supplementary Fig.1

Typical image showing *Calluna vulgaris* collected from isolation site (A). Effect of *Bacillus velezensis* CBE on *B. distachyon* growth under different salt concentration (B)

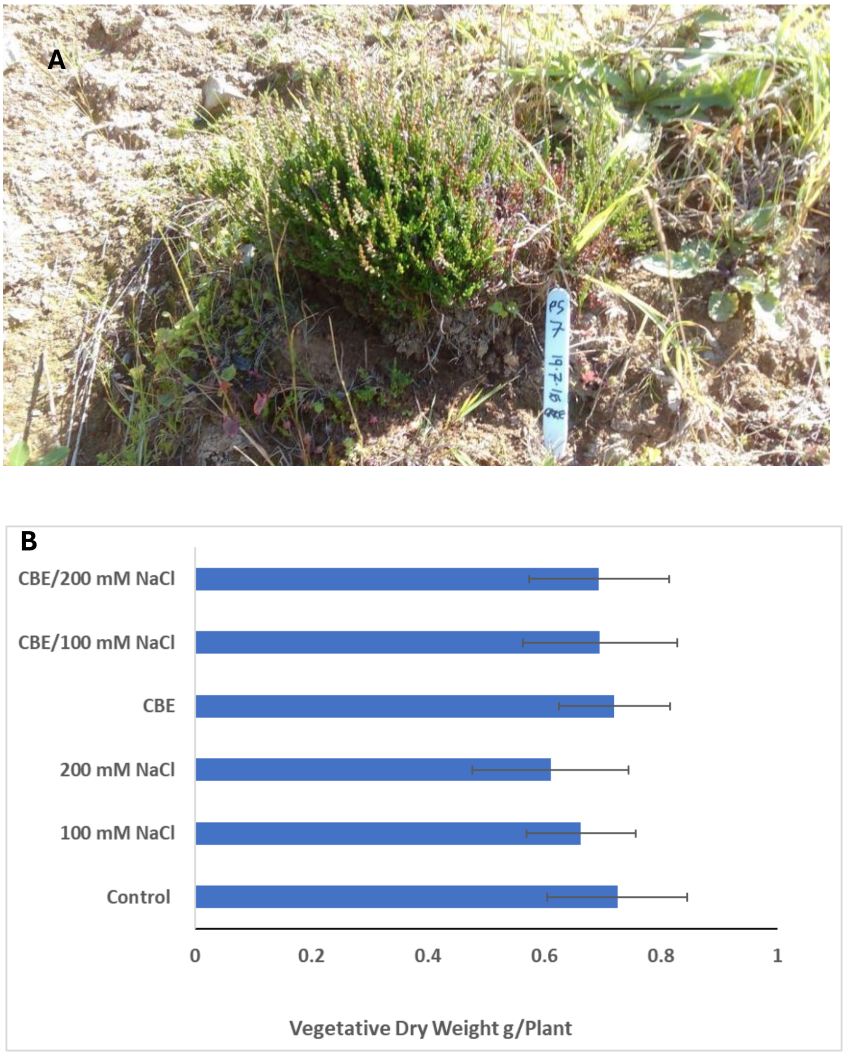

Supplement: Supplementary file 1 [file DataSheet1.pdf]
