# Supplementary material for: Halotolerant bacterial endophyte Bacillus velezensis CBE mediates abiotic stress tolerance with minimal transcriptional modifications in Brachypodium distachyon
Source: Front Plant Sci. 2025 Jan 10;15:1485391. doi: 10.3389/fpls.2024.1485391 (PMC11757260; doi:10.3389/fpls.2024.1485391)
Supplement: Supplementary file 2 [file DataSheet2.pdf]

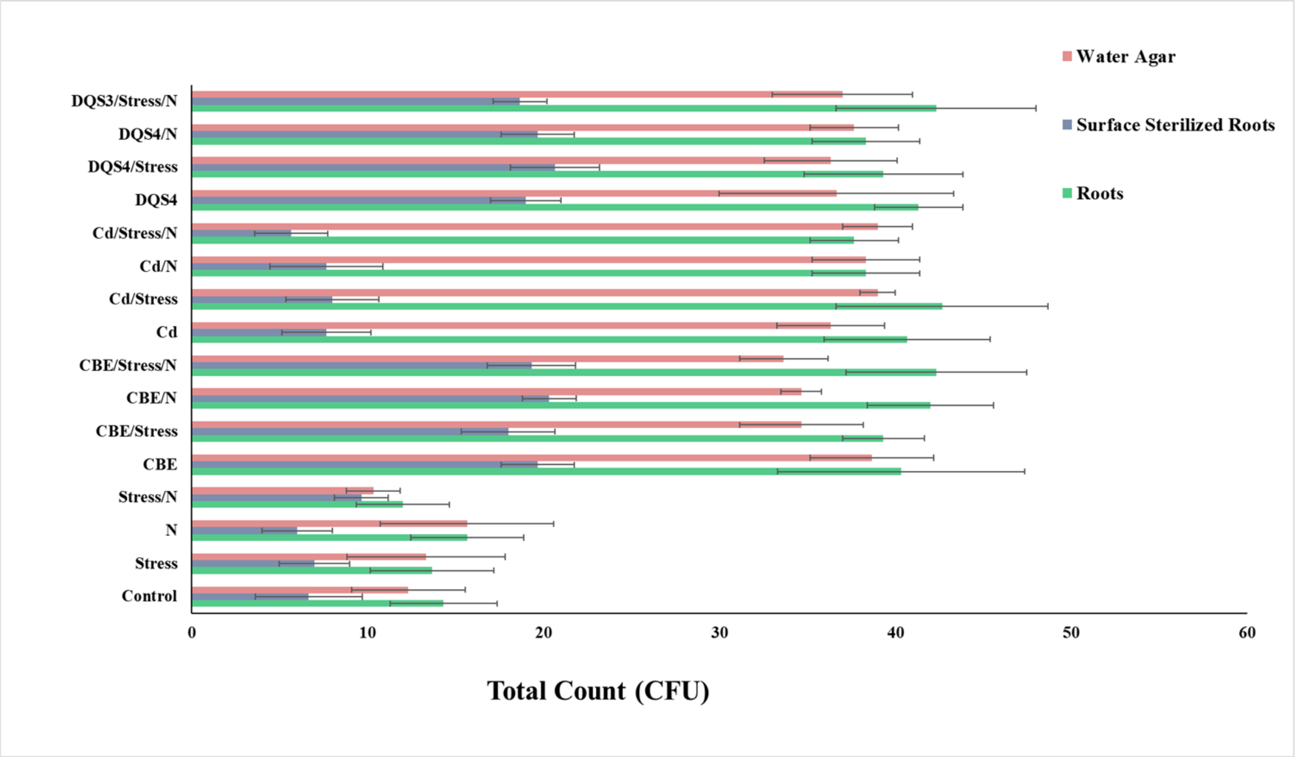

Supplementary Fig.2

Bacterial total counts on TSA plates. Represented as Colony forming units (CFU) per 1 ml media or 100 mg plant tissue (roots or surface sterilized roots). Bars indicate standard deviation (n=3).
