# Supplementary material for: Halotolerant bacterial endophyte Bacillus velezensis CBE mediates abiotic stress tolerance with minimal transcriptional modifications in Brachypodium distachyon
Source: Front Plant Sci. 2025 Jan 10;15:1485391. doi: 10.3389/fpls.2024.1485391 (PMC11757260; doi:10.3389/fpls.2024.1485391)
Supplement: Supplementary file 3 [file DataSheet3.pdf]

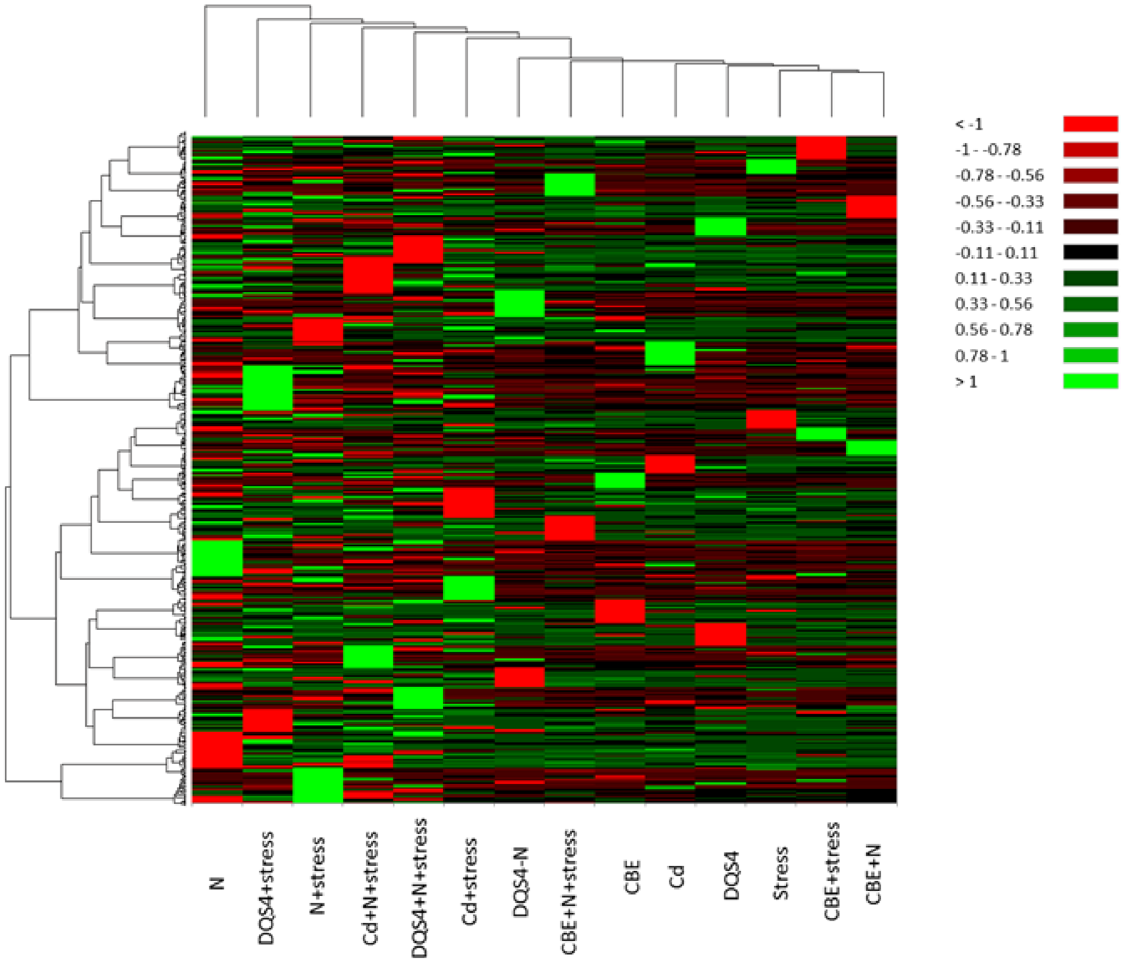

Supplementary Fig.3

RNA-seq showing differential genes expression in 18 days old *B. distachyon* Bd2 seedlings after treatment with novel halotolerant endophyte (*B. velezensis* CBE), rhizospheric control strain (*A. brasilense* Cd) or endophytic control strain (*A. Olearius* DQS4). Seedlings were grown for 14 days either in sterile water agar (2.5 g/ml) (control) or in 5% PEG8000 (osmotic stress) under normal nitrogen supply (3mM NH_4NO_3) or no nitrogen supply. Heat map represent the average fold expression of three biological samples were generated based on Pearson and Ward for distance measure and clustering using XLSTAT package.
