# Supplementary material for: Halotolerant bacterial endophyte Bacillus velezensis CBE mediates abiotic stress tolerance with minimal transcriptional modifications in Brachypodium distachyon
Source: Front Plant Sci. 2025 Jan 10;15:1485391. doi: 10.3389/fpls.2024.1485391 (PMC11757260; doi:10.3389/fpls.2024.1485391)
Supplement: Supplementary file 4 [file DataSheet4.pdf]

Supplementary methodology: Determining the ability of *Bacillus velezensis* CBE to tolerate salt stress

Salt stress tolerance of *B velezensis* CBE was tested along with other endophytes according to the protocol blew

1. Growth of liquid cultures

Endophytes were grown from long-term storage on nutrient agar at 28˚C for 48 hours. Subsequently 10ml nutrient broth was inoculated with a loopful of the endophyte and grown at 28˚C for 24 hours at 200rpm.

2. Growth on salt-supplemented agar

Square agar plates were prepared with Nutrient agar supplemented with either 0M, 1M or 2M additional NaCl. From each liquid culture 10µl was spotted carefully onto the agar surface and allowed to dry before turning the plate in order to ensure no spreading of the droplet. Plates were each inoculated with 9 endophytes in a 3x3 grid. Each set of endophytes was inoculated in triplicate, with each replicate randomly assorted to ensure random arrangements A, B, and C of the endophytes within the replicate. Each set of endophytes were inoculated within their assortments (A, B and C) onto each level of salt-supplemented agar plate. Thus for each pattern of nine endophytes, three plates were inoculated in that pattern with one plate of each of the three tested salt concentrations.

All plates were sealed with parafilm and incubated at 28˚C for one week.

3. Image capture

Images were captured of each plate at 48 and 168 hours into the experiment. Images were taken using a lightbox beneath the agar, and an exposure of ten seconds was used in order to eliminate strobing effects from the light box. Each plate was held in the same framing by use of a tripod clamped into identical orientation in order to eliminate differences between replicates based on parallax errors. Use of a tripod also enabled clear imaging despite the lengthy exposure. Included in each image was a standard 15cm ruler to be used as a measurement reference by the image analysis software.

4. Image analysis

Images were processed using the software ImageJ. Using the ruler included within each image, scale was set manually for eac image. For each image this scale was reset in order to eliminate minor inaccuracies between each image.

Using colour-based fuzzy selection via ImageJ's in built 'wand' tool, each colony was individually highlighted manually. This was checked by eye to ensure that no erroneous selections were made, as with several endophytes' colony colour approached a similar shade to the agar itself which on occasion led to incorrect areas being highlighted by the programme. Once the correct area was highlighted, ImageJ evaluated the number of pixels covered by the selection and using the scale set previously using the 15cm ruler calculated the area represented by each colony. *B velezensis* CBE related data is presented supplementary table 1
