# Supplementary material for: Halotolerant bacterial endophyte Bacillus velezensis CBE mediates abiotic stress tolerance with minimal transcriptional modifications in Brachypodium distachyon
Source: Front Plant Sci. 2025 Jan 10;15:1485391. doi: 10.3389/fpls.2024.1485391 (PMC11757260; doi:10.3389/fpls.2024.1485391)
Supplement: Supplementary file 5 [file DataSheet5.pdf]

Supplementary table 1

Effect of different salt concentrations on *Bacillus velezensis* CBE growth over time

**Salt concentration**	**Replicate**	**Colony surface area (cm^2)**		**Colony volume Increase over time (cm^2)**	**Increase%**
		**48h**	**168h**		
0M	a	4.756	9.98	5.224	109.84
0M	b	3.283	7.44	4.157	126.62
0M	c	2.958	3.891	0.933	31.54
1M	a	0.421	1.133	0.712	169.12
1M	b	0.499	1.274	0.775	155.31
1M	c	0.488	1.332	0.844	172.95
2M	a	0.074	0.571	0.497	671.62
2M	b	0.041	0.459	0.418	1019.51
2M	c	0.12	0.546	0.426	355
