# Supplementary material for: Halotolerant bacterial endophyte Bacillus velezensis CBE mediates abiotic stress tolerance with minimal transcriptional modifications in Brachypodium distachyon
Source: Front Plant Sci. 2025 Jan 10;15:1485391. doi: 10.3389/fpls.2024.1485391 (PMC11757260; doi:10.3389/fpls.2024.1485391)
Supplement: Supplementary file 6 [file DataSheet6.pdf]

Supplementary

Supplementary table 2: List of genes with significant (p<0.01) differential expression in CBE treated seedlings grown for 14 days on nitrogen free water agar.

GENE	LFC	FUNCTION (PHYTOZOME bd21.v3)
Bradi3g27277.1	4.174116477	4.1.1.39 - Ribulose-bisphosphate carboxylase / RuBP carboxylase (1 of 11)
Bradi4g02110.1	2.742725451	NA
Bradi1g38238.1	2.558497752	PTHR31194:SF0 - ETHYLENE-RESPONSIVE TRANSCRIPTION FACTOR CRF3-RELATED (1 of 6)
Bradi2g11300.1	2.181539806	1.11.1.7 - Peroxidase / Lactoperoxidase (1 of 144)
Bradi2g16120.1	2.097977317	MYB-related transcription factor
Bradi3g10470.1	2.011656551	PTHR31235:SF70 - PEROXIDASE 60 (1 of 2)
Bradi1g07970.1	1.991416647	WRKY transcription factor
Bradi2g52405.1	1.872174415	NA
Bradi5g13110.1	1.758406359	PF02469 - Fasciclin domain (Fasciclin) (1 of 24)
Bradi1g20010.1	1.731136157	1.11.1.7 - Peroxidase / Lactoperoxidase (1 of 144)
Bradi2g38690.1	1.728493751	1.11.1.7 - Peroxidase / Lactoperoxidase (1 of 144)
Bradi1g51990.1	1.697873347	PF03330 - Rare lipoprotein A (RlpA)-like double-psi beta-barrel (DPBB_1) (1 of 60)
Bradi3g32110.1	1.694165079	1.11.1.7 - Peroxidase / Lactoperoxidase (1 of 144)
Bradi1g44565.1	1.665712161	NA
Bradi1g43887.1	1.642583558	PF01657//PF07714 - Salt stress response/antifungal (Stress-antifung) // Protein tyrosine kinase (Pkinase_Tyr) (1 of 26)
Bradi3g11140.1	1.624428084	NA
Bradi1g07942.1	1.619649832	PF07714//PF11721 - Protein tyrosine kinase (Pkinase_Tyr) // Di-glucose binding within endoplasmic reticulum (Malectin) (1 of 12)
Bradi3g09005.1	1.58698102	PF00069//PF01453 - Protein kinase domain (Pkinase) // D-mannose binding lectin (B_lectin) (1 of 10)
Bradi1g51554.1	1.528614999	NA
Bradi1g14800.1	1.476596402	NA
Bradi1g59520.1	1.454342045	1.11.1.7 - Peroxidase / Lactoperoxidase (1 of 144)
Bradi3g08280.1	1.446011781	bHLH transcription factor
Bradi1g36566.1	1.428871633	NA
Bradi1g26870.1	1.42860938	PTHR31235:SF8 - PEROXIDASE 7 (1 of 1)
Bradi2g03930.1	1.399351	PF01190 - Pollen proteins Ole e I like (Pollen_Ole_e_I) (1 of 27)
Bradi5g11830.1	1.371300137	aminopeptidase activity, cysteine-type endopeptidase activity
Bradi2g45480.1	1.340913193	WRKY transcription factor
Bradi3g31767.1	1.337153932	K08235 - xyloglucan:xyloglucosyl transferase (E2.4.1.207) (1 of 22)
Bradi1g64300.1	1.335434325	PF03140 - Plant protein of unknown function (DUF247) (1 of 56)
Bradi1g42516.1	1.334872003	NA
Bradi3g28417.1	1.326944372	PTHR11654:SF73 - PROTEIN NRT1/ PTR FAMILY 5.2-RELATED (1 of 2)
Bradi2g38685.1	1.31924084	1.11.1.7 - Peroxidase / Lactoperoxidase (1 of 144)
Bradi1g25480.1	1.268118903	copper ion binding
Bradi1g08145.1	1.252834883	PTHR11926//PTHR11926:SF361 - GLUCOSYL/GLUCURONOSYL TRANSFERASES // SUBFAMILY NOT NAMED (1 of 2)
Bradi3g39980.1	1.226007008	inorganic diphosphatase activity
Bradi3g46040.1	1.222736969	RING, subfamily zinc finger (C3HC4-type RING finger) family protein
Bradi3g58180.1	1.210650826	N-methyltransferase activity
Bradi3g03650.1	1.192214425	NA
Bradi1g41115.1	1.18824805	1.11.1.7 - Peroxidase / Lactoperoxidase (1 of 144)
Bradi1g42513.1	1.169111143	NA
Bradi1g37930.1	1.166701383	NA
Bradi1g72230.1	1.145241218	PF02458 - Transferase family (Transferase) (1 of 99)

Bradi3g33130.1	1.129766331	PF03330 - Rare lipoprotein A (RlpA)-like double-psi beta-barrel (DPBB_1) (1 of 60)
Bradi3g10480.1	1.122388841	AGC_PVPK_like_kin82y.6 - AGC kinases include homologs to PKA, PKG and PKC, subfamily PVPK_like(AGC_8)_kin82y
Bradi3g60756.1	1.089970846	PF00083//PF07690 - Sugar (and other) transporter (Sugar_tr) // Major Facilitator Superfamily (MFS_1) (1 of 8)
Bradi4g39960.1	1.071513266	zinc ion binding, alcohol dehydrogenase activity
Bradi2g48940.1	1.06059222	NA
Bradi1g20020.1	1.013713101	1.11.1.7 - Peroxidase / Lactoperoxidase (1 of 144)
Bradi3g38310.1	1.00896747	NA
Bradi3g42627.1	1.007859067	NA
Bradi3g50190.1	-1.004540091	KOG0617 - Ras suppressor protein (contains leucine-rich repeats) (1 of 12)
Bradi1g63220.1	-1.008476712	WRKY transcription factor
Bradi2g00390.1	-1.009015548	NA
Bradi1g75607.1	-1.011350673	PTHR10579//PTHR10579:SF51 - CALCIUM-ACTIVATED CHLORIDE CHANNEL REGULATOR // SUBFAMILY NOT NAMED (1 of 6)
Bradi2g56850.1	-1.016492035	PTHR31155:SF3 - ACYL-[ACYL-CARRIER-PROTEIN] DESATURASE 6, CHLOROPLASTIC (1 of 1)
Bradi4g21980.1	-1.020897558	protein kinase family protein, putative, expressed, subfamily RLCK-OS2
Bradi2g10320.1	-1.033528265	PF03330 - Rare lipoprotein A (RlpA)-like double-psi beta-barrel (DPBB_1) (1 of 60)
Bradi1g17140.1	-1.044594129	NA
Bradi3g04585.1	-1.049415641	PF12609 - Wound-induced protein (DUF3774) (1 of 11)
Bradi5g22650.1	-1.050019663	NA
Bradi5g12920.1	-1.054804047	NA
Bradi1g64550.1	-1.056644261	bZIP transcription factor
Bradi3g26850.1	-1.057315	Glycosyl hydrolase (GH), subfamily GH18
Bradi3g28995.1	-1.057792611	NA
Bradi2g18920.1	-1.061147141	PTHR33470:SF4 - ARABINOGALACTAN PROTEIN 31 (1 of 1)
Bradi1g42900.1	-1.061945292	1.11.1.7 - Peroxidase / Lactoperoxidase (1 of 144)
Bradi1g13868.1	-1.070844549	NA
Bradi4g27930.1	-1.072854091	NA
Bradi5g09610.1	-1.07744936	PF14368 - Probable lipid transfer (LTP_2) (1 of 72)
Bradi2g12216.1	-1.083042024	1.11.1.7 - Peroxidase / Lactoperoxidase (1 of 144)
Bradi4g39310.1	-1.086769881	ATP binding
Bradi1g25517.1	-1.091050926	3.2.1.39 - Glucan endo-1,3-beta-D-glucosidase / Laminarinase (1 of 61)
Bradi2g10340.1	-1.092143365	MYB transcrition factor
Bradi3g28006.1	-1.095807203	PTHR11062//PTHR11062:SF111 - EXOSTOSIN HEPARAN SULFATE GLYCOSYLTRANSFERASE -RELATED // SUBFAMILY NOT NAMED (1 of 1)
Bradi3g07770.1	-1.098210047	UDP-galactosyltransferase activity
Bradi1g24340.1	-1.104457989	sterol 14-demethylase activity
Bradi3g03070.1	-1.106500542	PF07816 - Protein of unknown function (DUF1645) (DUF1645) (1 of 9)
Bradi1g53260.1	-1.111179653	PF03168 - Late embryogenesis abundant protein (LEA_2) (1 of 62)
Bradi1g71990.1	-1.112615186	NA
Bradi3g45636.1	-1.115361947	Glycosyl hydrolase (GH), subfamily GH1
Bradi2g45500.1	-1.122189892	NA
Bradi2g11890.1	-1.122746141	PF00847 - AP2 domain (AP2) (1 of 161)
Bradi2g22241.1	-1.126270977	PF03106 - WRKY DNA -binding domain (WRKY) (1 of 89)
Bradi4g25660.1	-1.128187292	PTHR31388:SF24 - PEROXIDASE 52 (1 of 4)
Bradi5g10170.1	-1.131039501	K02947 - small subunit ribosomal protein S10e (RP-S10e, RPS10) (1 of 3)
Bradi3g04671.1	-1.134984969	PTHR10795//PTHR10795:SF365 - PROPROTEIN CONVERTASE SUBTILISIN/KEXIN // SUBFAMILY NOT NAMED (1 of 3)
Bradi4g05540.1	-1.14295191	ion channel activity
Bradi4g06740.1	-1.145883448	PF07168 - Ureide permease (Ureide_permease) (1 of 4)
Bradi2g26000.1	-1.152113382	Glycosyl hydrolase (GH), subfamily GH19
Bradi3g27840.1	-1.15352751	NA
Bradi2g48680.1	-1.160434865	PF01190 - Pollen proteins Ole e I like (Pollen_Ole_e_I) (1 of 27)

Bradi1g38310.1	-1.162777878	1.11.1.7 - Peroxidase / Lactoperoxidase (1 of 144)
Bradi4g11241.1	-1.167735567	NA
Bradi1g17860.1	-1.16805375	1.11.1.7 - Peroxidase / Lactoperoxidase (1 of 144)
Bradi5g19680.1	-1.176657711	CrRLK1L
Bradi1g43130.1	-1.178179197	NA
Bradi4g23800.1	-1.178657537	NA
Bradi2g36606.1	-1.18212035	NA
Bradi4g09680.1	-1.182246407	NA
Bradi3g03170.1	-1.183968088	PF00564//PF02042 - PB1 domain (PB1) // RWP-RK domain (RWP-RK) (1 of 7)
Bradi3g33140.1	-1.184663323	PF03330 - Rare lipoprotein A (RlpA)-like double-psi beta-barrel (DPBB_1) (1 of 60)
Bradi4g37470.1	-1.186765634	estrone sulfotransferase activity
Bradi2g45030.1	-1.187997734	NA
Bradi3g21806.1	-1.193047139	NA
Bradi1g76430.1	-1.195141456	AMP binding
Bradi2g44160.1	-1.197292489	testosterone 6-beta-hydroxylase activity
Bradi1g11680.1	-1.197707633	iron ion binding, lipoxygenase activity
Bradi1g22710.1	-1.202362474	cysteine protease inhibitor activity
Bradi3g00460.1	-1.222018798	NA
Bradi3g07000.1	-1.22494675	NA
Bradi1g25447.1	-1.227872685	NA
Bradi4g09727.1	-1.230393063	NA
Bradi1g55830.1	-1.232581386	PF14009 - Domain of unknown function (DUF4228) (DUF4228) (1 of 38)
Bradi5g14470.1	-1.235757548	PF04862 - Protein of unknown function (DUF642) (DUF642) (1 of 9)
Bradi5g21720.1	-1.239231007	PF04862 - Protein of unknown function (DUF642) (DUF642) (1 of 9)
Bradi2g10294.1	-1.239501681	1.14.13.137 - Indole-2-monooxygenase / CYP71C4 (1 of 6)
Bradi2g25280.1	-1.240704947	protein kinase family protein, putative, expressed, subfamily SD-2b
Bradi3g29030.1	-1.242679832	K07192 - flotillin (FLOT) (1 of 3)
Bradi1g67080.1	-1.249734123	PTHR11527//PTHR11527:SF152 - SMALL HEAT-SHOCK PROTEIN HSP20 FAMILY // SUBFAMILY NOT NAMED (1 of 6)
Bradi3g43160.1	-1.252078749	steroid 17-alpha-monooxygenase activity
Bradi3g10510.1	-1.257303169	NA
Bradi2g58989.1	-1.261193802	PF03407 - Nucleotide-diphospho-sugar transferase (Nucleotid_trans) (1 of 26)
Bradi5g01900.1	-1.271981297	bHLH transcription factor
Bradi4g11204.1	-1.27206328	NA
Bradi1g26215.1	-1.281001334	1.11.1.7 - Peroxidase / Lactoperoxidase (1 of 144)
Bradi2g51170.1	-1.286605502	histidine decarboxylase activity, aromatic-L-amino-acid decarboxylase activity, sulfinoalanine decarboxylase activity
Bradi2g27220.1	-1.296188654	protein kinase activity
Bradi3g60100.1	-1.297373622	PTHR11527:SF106 - 17.4 KDA CLASS III HEAT SHOCK PROTEIN (1 of 1)
Bradi1g22860.1	-1.308877537	steroid dehydrogenase activity, acting on the CH-OH group of donors, NAD or NADP as acceptor
Bradi1g19470.1	-1.309685905	PF14368 - Probable lipid transfer (LTP_2) (1 of 72)
Bradi1g48292.1	-1.31097977	PTHR21495//PTHR21495:SF76 - NUCLEOPORIN-RELATED // SUBFAMILY NOT NAMED (1 of 5)
Bradi1g71500.1	-1.312721976	NA
Bradi4g32941.1	-1.314772625	K09487 - heat shock protein 90kDa beta (HSP90B, TRA1) (1 of 4)
Bradi3g47080.1	-1.319520586	NA
Bradi3g45643.1	-1.320567277	PTHR10353//PTHR10353:SF50 - GLYCOSYL HYDROLASE // SUBFAMILY NOT NAMED (1 of 8)
Bradi2g14970.1	-1.326760963	ADP-Glucose pyrophosphorylase, large subunit
Bradi1g32280.1	-1.327655681	K13065 - shikimate O-hydroxycinnamoyltransferase (E2.3.1.133, HCT) (1 of 7)
Bradi4g09740.1	-1.332266471	chitinase activity
Bradi4g12814.1	-1.333124778	NA
Bradi1g53850.1	-1.333868122	PTHR11527//PTHR11527:SF152 - SMALL HEAT-SHOCK PROTEIN HSP20 FAMILY // SUBFAMILY NOT NAMED (1 of 6)

Bradi1g26530.1	-1.335909019	cysteine-type endopeptidase activity, phospholipase C activity
Bradi1g58210.1	-1.336683699	aldo-keto reductase activity
Bradi1g19713.1	-1.34154835	PTHR23024//PTHR23024:SF152 - MEMBER OF 'GDXG' FAMILY OF LIPOLYTIC ENZYMES // SUBFAMILY NOT NAMED (1 of 3)
Bradi2g45610.1	-1.343618998	Glycosyl hydrolase (GH), subfamily GH18
Bradi3g15940.1	-1.343762559	NA
Bradi3g35660.1	-1.350528242	voltage-gated calcium channel activity
Bradi2g52881.1	-1.356878801	PTHR33624:SF2 - SIGMA FACTOR BINDING PROTEIN 1, CHLOROPLASTIC-RELATED (1 of 2)
Bradi3g26640.1	-1.359036475	NA
Bradi2g00381.1	-1.362873675	NA
Bradi1g76110.1	-1.365434903	1.14.13.88 - Flavonoid 3',5'-hydroxylase / F3'5'H (1 of 3)
Bradi1g61530.1	-1.366231204	1.11.1.7 - Peroxidase / Lactoperoxidase (1 of 144)
Bradi1g37080.1	-1.378998701	histone acetyltransferase activity
Bradi3g21747.1	-1.396442884	PTHR23024//PTHR23024:SF231 - MEMBER OF 'GDXG' FAMILY OF LIPOLYTIC ENZYMES // SUBFAMILY NOT NAMED (1 of 6)
Bradi3g31720.1	-1.398751787	PTHR11260//PTHR11260:SF264 - GLUTATHIONE S-TRANSFERASE, GST, SUPERFAMILY, GST DOMAIN CONTAINING // SUBFAMILY NOT NAMED (1 of 4)
Bradi3g32770.1	-1.412867089	NA
Bradi3g47110.1	-1.429588672	Phenylalanine ammonia lyase (PAL)
Bradi1g44570.1	-1.433923954	NA
Bradi2g27920.1	-1.434476508	PTHR31190:SF21 - ETHYLENE-RESPONSIVE TRANSCRIPTION FACTOR 13 (1 of 1)
Bradi3g15210.1	-1.438699845	manganese ion binding
Bradi5g11950.1	-1.450473773	NA
Bradi1g59537.1	-1.457476795	1.11.1.7 - Peroxidase / Lactoperoxidase (1 of 144)
Bradi2g39290.1	-1.463549975	trypsin inhibitor activity
Bradi1g43860.1	-1.4676826	copper ion binding
Bradi5g03530.1	-1.472759146	NA
Bradi3g60280.1	-1.479092907	ion channel activity
Bradi3g36540.1	-1.500541199	triacylglycerol lipase activity
Bradi2g16790.1	-1.503144077	PTHR22952:SF210 - DOG1 ALPHA SPLICE VARIANT (1 of 1)
Bradi2g45053.1	-1.504866636	NA
Bradi1g46050.1	-1.510439769	Similar to fucosyltransferase CAZy family GT37
Bradi1g25130.1	-1.512471083	cellulose synthase-like (CSL), subfamily F
Bradi2g19690.1	-1.522412222	PTHR22924//PTHR22924:SF54 - LEGHEMOGLOBIN-RELATED // SUBFAMILY NOT NAMED (1 of 1)
Bradi3g41620.1	-1.525887427	NA
Bradi2g52210.1	-1.530995599	PTHR11474//PTHR11474:SF33 - TYROSINASE // SUBFAMILY NOT NAMED (1 of 1)
Bradi3g10030.1	-1.531510805	serine-type endopeptidase activity
Bradi3g09910.1	-1.532605199	NA
Bradi3g34320.1	-1.548615014	PTHR26312:SF53 - CARBOXYLATE CLAMP-TETRATRICOPEPTIDE REPEAT PROTEIN (1 of 1)
Bradi1g13760.1	-1.548871767	9-cis-epoxycarotenoid dioxygenase-like
Bradi4g09690.1	-1.563523869	chitinase activity
Bradi5g27330.1	-1.567763283	NA
Bradi3g34420.1	-1.568649333	NA
Bradi4g36968.1	-1.57581442	PTHR11926//PTHR11926:SF118 - GLUCOSYL/GLUCURONOSYL TRANSFERASES // SUBFAMILY NOT NAMED (1 of 4)
Bradi2g08760.1	-1.575854969	PF03330 - Rare lipoprotein A (RlpA)-like double-psi beta-barrel (DPBB_1) (1 of 60)
Bradi3g53681.1	-1.582186794	PTHR10334:SF207 - CAP (CYSTEINE-RICH SECRETORY PROTEINS, ANTIGEN 5, AND PATHOGENESIS-RELATED 1 PROTEIN) SUPERFAMILY PROTEIN (1 of 1)
Bradi1g30050.1	-1.582423082	PTHR31692:SF10 - EXPANSIN-LIKE B1 (1 of 1)
Bradi2g55270.1	-1.600814799	PF00046 - Homeobox domain (Homeobox) (1 of 64)
Bradi3g35480.1	-1.601441647	PTHR13871 - THIOREDOXIN (1 of 9)
Bradi1g51080.1	-1.610168792	PF03386 - Early nodulin 93 ENOD93 protein (ENOD93) (1 of 3)
Bradi5g15246.1	-1.615484075	PTHR10641:SF456 - MYB DOMAIN PROTEIN 79 (1 of 2)

Bradi3g04490.1	-1.633310208	PTHR13780:SF51 - SNF1-RELATED PROTEIN KINASE REGULATORY SUBUNIT GAMMA-LIKE PV42A-RELATED (1 of 1)
Bradi1g50390.1	-1.635902493	NA
Bradi5g03933.1	-1.668423764	NA
Bradi1g64350.1	-1.670292216	NA
Bradi2g32270.1	-1.682016582	Glycosyl hydrolase (GH), subfamily GH9
Bradi3g15809.1	-1.6972219	PTHR11019:SF100 - PROTEIN DJ-1 (1 of 2)
Bradi1g65590.1	-1.698910958	iron ion binding
Bradi5g09020.1	-1.702809383	prephenate dehydratase activity
Bradi5g07190.1	-1.708997028	2.2.1.1 - Transketolase / Glycoaldehyde transferase (1 of 2)
Bradi1g48160.1	-1.714397432	PF14547 - Hydrophobic seed protein (Hydrophob_seed) (1 of 20)
Bradi4g18970.1	-1.714603151	FAD binding
Bradi2g25460.1	-1.720401712	proton-dependent oligopeptide secondary active transmembrane transporter activity
Bradi2g45470.1	-1.728916254	protein serine/threonine phosphatase activity
Bradi1g13070.1	-1.740701982	PF00314 - Thaumatin family (Thaumatin) (1 of 34)
Bradi3g29710.1	-1.744631346	PF04450 - Peptidase of plants and bacteria (BSP) (1 of 3)
Bradi3g02290.1	-1.749533759	copper ion binding
Bradi3g46680.1	-1.754927111	K14514 - ethylene-insensitive protein 3 (EIN3) (1 of 6)
Bradi2g41840.1	-1.757757892	iron ion binding
Bradi3g59718.1	-1.761176779	PTHR22835//PTHR22835:SF190 - ZINC FINGER FYVE DOMAIN CONTAINING PROTEIN // SUBFAMILY NOT NAMED (1 of 5)
Bradi2g45360.1	-1.780172605	PF14416 - PMR5 N terminal Domain (PMR5N) (1 of 47)
Bradi1g31337.1	-1.781539825	K09285 - AP2-like factor, ANT lineage (OVM, ANT) (1 of 17)
Bradi5g27340.1	-1.786961803	PF08263 - Leucine rich repeat N-terminal domain (LRRNT_2) (1 of 251)
Bradi1g63510.1	-1.811823403	phosphoglycerate mutase activity, manganese ion binding
Bradi3g33780.1	-1.827939992	1.11.1.7 - Peroxidase / Lactoperoxidase (1 of 144)
Bradi2g27680.1	-1.838065339	PF15699 - NPR1 interacting (NPR1_interact) (1 of 3)
Bradi3g59700.1	-1.861831356	NA
Bradi2g60190.1	-1.863792754	aspartic-type endopeptidase activity
Bradi3g35670.1	-1.86628422	PF07676 - WD40-like Beta Propeller Repeat (PD40) (1 of 8)
Bradi3g26130.1	-1.870760166	NA
Bradi2g35500.1	-1.873280971	trypsin inhibitor activity
Bradi1g47210.1	-1.886456922	NA
Bradi5g03180.1	-1.891009202	WAK receptor-like protein kinase, subfamily WAKL-OS
Bradi3g26638.1	-1.903000586	NA
Bradi2g36612.1	-1.90489024	NA
Bradi2g11567.1	-1.905145007	PTHR15371//PTHR15371:SF5 - TIM23 // SUBFAMILY NOT NAMED (1 of 1)
Bradi1g78772.1	-1.93430696	NA
Bradi4g14620.1	-1.93565266	PF04398 - Protein of unknown function, DUF538 (DUF538) (1 of 35)
Bradi1g09270.1	-1.959514162	iron ion binding, lipoxygenase activity
Bradi4g10465.1	-1.959892602	NA
Bradi5g09270.1	-1.969387847	Homologous to Arabidopsis CENTRORADIALIS
Bradi2g52510.1	-1.990117286	acid phosphatase activity
Bradi2g58350.1	-1.997916874	NA
Bradi5g02037.1	-1.998993264	PTHR11528:SF53 - HEAT SHOCK PROTEIN 90-1 (1 of 1)
Bradi1g14580.1	-2.008774272	iron ion binding
Bradi4g12011.1	-2.010153039	NA
Bradi2g04252.1	-2.019413337	PTHR10641//PTHR10641:SF663 - MYB-LIKE DNA-BINDING PROTEIN MYB // SUBFAMILY NOT NAMED (1 of 2)
Bradi2g51440.1	-2.022446298	serine-type endopeptidase activity
Bradi3g02810.1	-2.109994429	NA
Bradi1g75110.1	-2.136472652	PTHR31529:SF2 - LOB DOMAIN-CONTAINING PROTEIN 17-RELATED (1 of 1)

Bradi3g22215.1	-2.176820316	NA
Bradi4g44027.1	-2.190210093	K14641 - apyrase (APY1_2) (1 of 5)
Bradi5g25021.1	-2.196954852	NA
Bradi3g02590.1	-2.213263717	NA
Bradi2g54222.1	-2.213320945	NA
Bradi3g47120.1	-2.240486154	Phenylalanine ammonia lyase (PAL)
Bradi3g20160.1	-2.256613704	ATP-dependent DNA helicase activity
Bradi3g12570.1	-2.259113431	NA
Bradi4g39520.1	-2.268973375	PTHR33595:SF3 - EMB (1 of 1)
Bradi2g00720.1	-2.278601362	bHLH transcription factor
Bradi4g12340.1	-2.279230607	PTHR21495//PTHR21495:SF81 - NUCLEOPORIN-RELATED // SUBFAMILY NOT NAMED (1 of 6)
Bradi1g77130.1	-2.280215704	1.11.1.7 - Peroxidase / Lactoperoxidase (1 of 144)
Bradi3g46190.1	-2.287857518	PF03242 - Late embryogenesis abundant protein (LEA_3) (1 of 7)
Bradi1g26448.1	-2.304608626	NA
Bradi4g12350.1	-2.307709519	chloride channel activity
Bradi2g47072.1	-2.314795283	NA
Bradi3g57680.1	-2.332871707	AMP binding, ADP binding
Bradi4g35214.1	-2.349324515	NA
Bradi2g61600.1	-2.355051728	PF02496 - ABA/WDS induced protein (ABA_WDS) (1 of 5)
Bradi2g35450.1	-2.361630369	triacylglycerol lipase activity
Bradi3g03556.1	-2.377761234	NA
Bradi4g13580.1	-2.399398874	PF01117 - Aerolysin toxin (Aerolysin) (1 of 2)
Bradi5g23546.1	-2.3998842	NA
Bradi4g34530.1	-2.423119155	Similar to glycosyltransferase. CAZy family GT77
Bradi2g09600.1	-2.430068736	1.11.1.7 - Peroxidase / Lactoperoxidase (1 of 144)
Bradi2g36620.1	-2.437767692	NA
Bradi1g33075.1	-2.453996079	NA
Bradi2g61607.1	-2.468819935	PF02496 - ABA/WDS induced protein (ABA_WDS) (1 of 5)
Bradi3g02310.1	-2.476361475	2.3.1.64 - Agmatine N(4)-coumaroyltransferase / p-coumaroyl-CoA-agmatine N-p-coumaroyltransferase (1 of 6)
Bradi2g07150.1	-2.505665007	Glycosyl hydrolase (GH), subfamily GH9
Bradi4g14640.1	-2.516083209	PF04398 - Protein of unknown function, DUF538 (DUF538) (1 of 35)
Bradi1g15695.1	-2.586465302	1.14.13.41 - Tyrosine N-monooxygenase / Tyrosine N-hydroxylase (1 of 5)
Bradi5g27150.1	-2.598773804	1.11.1.7 - Peroxidase / Lactoperoxidase (1 of 144)
Bradi5g27170.1	-2.601055585	1.11.1.7 - Peroxidase / Lactoperoxidase (1 of 144)
Bradi4g09430.1	-2.616434285	Glycosyl hydrolase (GH), subfamily GH18
Bradi2g17530.1	-2.627016975	PF14368 - Probable lipid transfer (LTP_2) (1 of 72)
Bradi2g58340.1	-2.660378168	NA
Bradi3g51660.1	-2.662126564	KOG2325 - Predicted transporter/transmembrane protein (1 of 3)
Bradi2g15360.1	-2.684924536	WRKY transcription factor
Bradi2g47210.1	-2.777528457	Glycosyl hydrolase (GH), subfamily GH19
Bradi4g36870.1	-2.787438991	arginine decarboxylase activity, ornithine decarboxylase activity
Bradi5g02077.1	-2.807986055	PTHR22835//PTHR22835:SF190 - ZINC FINGER FYVE DOMAIN CONTAINING PROTEIN // SUBFAMILY NOT NAMED (1 of 5)
Bradi2g02410.1	-2.858173388	PTHR11527//PTHR11527:SF152 - SMALL HEAT-SHOCK PROTEIN HSP20 FAMILY // SUBFAMILY NOT NAMED (1 of 6)
Bradi2g49540.1	-2.868661375	malate dehydrogenase (oxaloacetate-decarboxylating) (NADP+) activity, malate dehydrogenase (oxaloacetate-decarboxylating) activity, malate dehydrogenase (decarboxylating) activity, ADP binding
Bradi2g02400.1	-2.915001884	PTHR11527//PTHR11527:SF152 - SMALL HEAT-SHOCK PROTEIN HSP20 FAMILY // SUBFAMILY NOT NAMED (1 of 6)
Bradi4g30360.1	-2.929510704	WRKY transcription factor
Bradi1g67040.1	-2.955043005	PTHR11527//PTHR11527:SF152 - SMALL HEAT-SHOCK PROTEIN HSP20 FAMILY // SUBFAMILY NOT NAMED (1 of 6)
Bradi5g14475.1	-2.988627627	NA

Bradi2g54090.1	-3.060843019	NA
Bradi4g07130.1	-3.0795089	aminopeptidase activity, cysteine-type endopeptidase activity
Bradi2g18090.1	-3.093810861	PTHR23241:SF44 - LATE EMBRYOGENESIS ABUNDANT DOMAIN-CONTAINING PROTEIN (1 of 4)
Bradi4g34520.1	-3.096242694	NA
Bradi2g22460.1	-3.171862577	bile acid transmembrane transporter activity
Bradi3g30320.1	-3.183386915	PTHR33836:SF1 - ARM REPEAT SUPERFAMILY PROTEIN-RELATED (1 of 1)
Bradi3g59714.1	-3.185463426	PTHR22835//PTHR22835:SF190 - ZINC FINGER FYVE DOMAIN CONTAINING PROTEIN // SUBFAMILY NOT NAMED (1 of 5)
Bradi4g11216.1	-3.189233484	NA
Bradi4g17200.1	-3.22254943	NA
Bradi1g73840.1	-3.239693532	PTHR23241//PTHR23241:SF63 - LATE EMBRYOGENESIS ABUNDANT PLANTS LEA-RELATED // SUBFAMILY NOT NAMED (1 of 1)
Bradi4g19525.1	-3.246650671	PTHR33346:SF3 - DEHYDRIN RAB18-RELATED (1 of 1)
Bradi1g60563.1	-3.261688009	PTHR33348:SF3 - C-TERMINALLY ENCODED PEPTIDE 1 (1 of 2)
Bradi1g73970.1	-3.439326873	PTHR36326:SF1 - PROTEIN MALE STERILE 5 (1 of 4)
Bradi5g00970.1	-3.466862419	PTHR31225:SF16 - 1,8-CINEOLE SYNTHASE 1, CHLOROPLASTIC-RELATED (1 of 1)
Bradi1g51800.1	-3.5330985	PTHR33493:SF6 - LEA D113 HOMOLOGUE TYPE2-RELATED (1 of 2)
Bradi4g39920.1	-3.53996375	IgA binding, mannose binding, glucose binding
Bradi1g17961.1	-3.559843611	K09286 - EREBP-like factor (EREBP) (1 of 27)
Bradi4g04965.1	-3.612518562	NA
Bradi1g57280.1	-3.626113346	PTHR33920:SF1 - THIONIN-2.1-RELATED (1 of 12)
Bradi1g57285.1	-3.663383578	PTHR33920:SF1 - THIONIN-2.1-RELATED (1 of 12)
Bradi2g18106.1	-3.66614515	PTHR23241:SF44 - LATE EMBRYOGENESIS ABUNDANT DOMAIN-CONTAINING PROTEIN (1 of 4)
Bradi3g35680.1	-3.685917719	PF07676 - WD40-like Beta Propeller Repeat (PD40) (1 of 8)
Bradi1g69320.1	-3.723960712	PTHR22924 - LEGHEMOGLOBIN-RELATED (1 of 2)
Bradi3g08810.1	-3.739313418	PTHR13935:SF46 - BASIC HELIX-LOOP-HELIX DOMAIN-CONTAINING PROTEIN (1 of 1)
Bradi5g10450.1	-3.975727257	PF06884 - Protein of unknown function (DUF1264) (DUF1264) (1 of 3)
Bradi3g19730.1	-3.985885854	NA
Bradi3g34727.1	-4.07368861	NA
Bradi1g00600.1	-4.103600739	PTHR33294:SF5 - AWPM-19-LIKE FAMILY PROTEIN (1 of 1)
Bradi1g30034.1	-4.244506656	PTHR22835//PTHR22835:SF190 - ZINC FINGER FYVE DOMAIN CONTAINING PROTEIN // SUBFAMILY NOT NAMED (1 of 5)
Bradi4g20770.1	-4.323591783	PF02458 - Transferase family (Transferase) (1 of 99)
Bradi1g51770.1	-4.485497826	PTHR33493:SF6 - LEA D113 HOMOLOGUE TYPE2-RELATED (1 of 2)
Bradi2g33170.1	-4.694569649	PF03760 - Late embryogenesis abundant (LEA) group 1 (LEA_1) (1 of 7)
Bradi1g20950.1	-4.727903497	PTHR21495//PTHR21495:SF64 - NUCLEOPORIN-RELATED // SUBFAMILY NOT NAMED (1 of 7)
Bradi3g02130.1	-4.747228048	K15086 - (3S)-linalool synthase (TPS14) (1 of 1)
Bradi1g57337.1	-4.756493305	PTHR33920:SF1 - THIONIN-2.1-RELATED (1 of 12)
Bradi3g35700.1	-4.786762996	voltage-gated calcium channel activity
Bradi5g23550.1	-4.912769281	pectin methylesterase (PME)
Bradi3g53601.1	-5.044479661	PTHR31579:SF15 - F2401.16 (1 of 1)
Bradi1g37410.1	-5.047501354	dehydrin-3
Bradi4g44342.1	-5.063991301	PF03106 - WRKY DNA -binding domain (WRKY) (1 of 89)
Bradi3g22515.1	-5.200785644	NA
Bradi1g37600.1	-5.245417396	L-ascorbate oxidase activity, copper ion binding
Bradi2g27280.1	-5.410730401	NA
Bradi4g22535.1	-6.893574009	NA
Bradi1g10567.1	-7.040401452	PTHR11709//PTHR11709:SF86 - MULTI-COPPER OXIDASE // SUBFAMILY NOT NAMED (1 of 3)
Bradi4g20520.1	-7.562234914	voltage-gated calcium channel activity
